# Single-cell transcriptomics reveals variations in monocytes and Tregs between gout flare and remission

**DOI:** 10.1172/jci.insight.171417

**Published:** 2023-12-08

**Authors:** Hanjie Yu, Wen Xue, Hanqing Yu, Yaxiang Song, Xinying Liu, Ling Qin, Shu Wang, Hui Bao, Hongchen Gu, Guangqi Chen, Dake Zhao, Yang Tu, Jiafen Cheng, Liya Wang, Zisheng Ai, Dayong Hu, Ling Wang, Ai Peng

**Affiliations:** 1 Center for Nephrology and Clinical Metabolomics and Division of Nephrology, Shanghai Tenth People’s Hospital, and; 2 Department of Medical Statistics, Tongji University School of Medicine, Shanghai, China.

**Keywords:** Immunology, Inflammation, Monocytes, T cells

## Abstract

Gout commonly manifests as a painful, self-limiting inflammatory arthritis. Nevertheless, the understanding of the inflammatory and immune responses underlying gout flares and remission remains ambiguous. Here, based on single-cell RNA-Seq and an independent validation cohort, we identified the potential mechanism of gout flare, which likely involves the upregulation of HLA-DQA1^+^ nonclassical monocytes and is related to antigen processing and presentation. Furthermore, Tregs also play an essential role in the suppressive capacity during gout remission. Cell communication analysis suggested the existence of altered crosstalk between monocytes and other T cell types, such as Tregs. Moreover, we observed the systemic upregulation of inflammatory and cytokine genes, primarily in classical monocytes, during gout flares. All monocyte subtypes showed increased arachidonic acid metabolic activity along with upregulation of prostaglandin-endoperoxide synthase 2 (PTGS2). We also detected a decrease in blood arachidonic acid and an increase in leukotriene B4 levels during gout flares. In summary, our study illustrates the distinctive immune cell responses and systemic inflammation patterns that characterize the transition from gout flares to remission, and it suggests that blood monocyte subtypes and Tregs are potential intervention targets for preventing recurrent gout attacks and progression.

## Graphical abstract

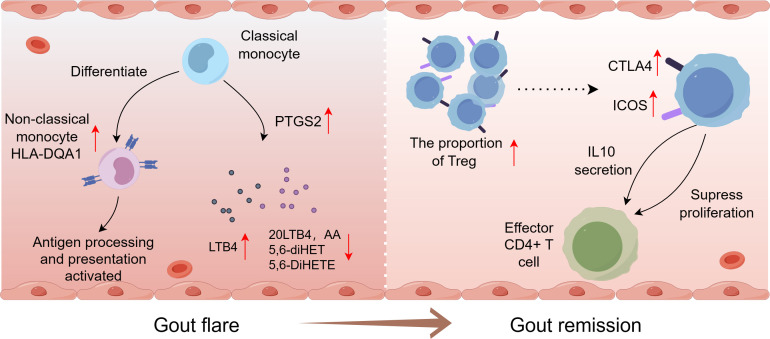

## Introduction

Gout is a complex and common arthritis that results from immune response dysfunction during sustained hyperuricemia, leading to the deposition and formation of monosodium urate (MSU) crystals (1). Acute gout flares predominantly affect the joints of the lower extremities (2). Gout flares typically undergo spontaneous remission for 1–2 weeks without treatment (2). Chronic hyperuricemia may promote frequent gout flares and remission. Hence, there is an urgent need to elucidate the mechanisms underlying the immune responses during the transition stage between gout flare episodes and remission.

Innate immune cells, such as blood monocytes and neutrophils, play a pivotal role in initiating and amplifying gout flares, which result from MSU deposition in the joints or tissues. This process leads to the release of the NOD-like Receptor Family Pyrin Domain Containing 3 (NLRP3) inflammasome and mediates the production of IL-1β (3–5). Moreover, chemokines, proinflammatory cytokines, and prostaglandin E2 have been implicated in the induction of inflammation associated with gout flares (6). Further research has highlighted the role of blood neutrophils in the resolution of gout flares through the formation of aggregated neutrophil extracellular traps (7), while recent in vitro studies have suggested that adaptive immunity is involved in both gout flares and remission (8–10). However, a comprehensive understanding of the inflammatory and immune responses during gout and its subsequent remission remains elusive.

Single-cell RNA-Seq (scRNA-Seq) has emerged as a powerful tool for generating unique gene expression profiles of individual cells, thereby elucidating their function and biological characteristics. In this study, we demonstrated how the landscape of blood immune cells changes between gout flares and remission, thereby offering valuable cell type–specific markers that could be utilized for potential targeted immune therapies.

## Results

### Single-cell transcriptome profiling of peripheral blood mononuclear cells (PBMCs) from gout flare and remission.

**Figure 1:**
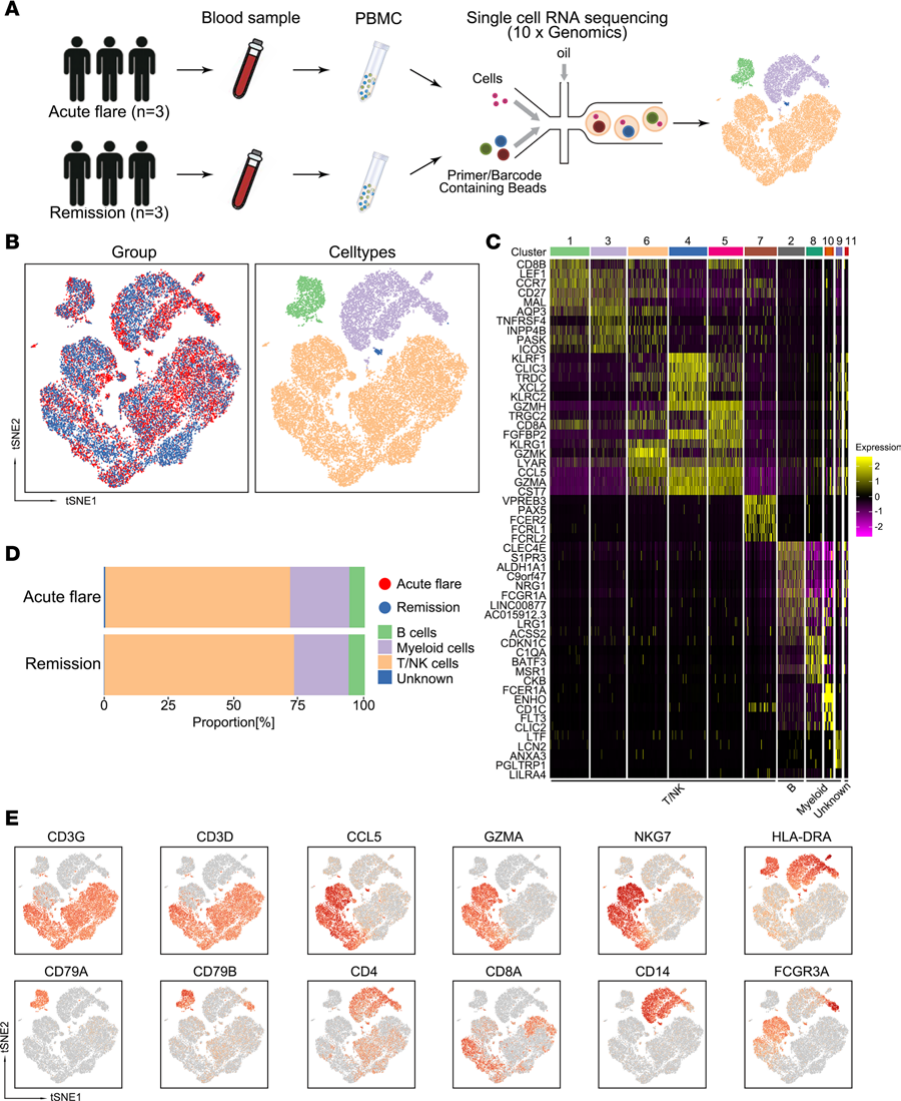
Single-cell transcriptome profiling of PBMCs between gout flare and remission. (**A**) Flowchart of the overall experiment design. Two paired peripheral blood samples (6 in total) were collected from each of the same 3 patients with gout flare (1 sample/patient), and later during gout remission (1 sample/patient). The samples were dissociated into single cells and sorted for scRNA-Seq. (**B**) The t-SNE representations of integrated single-cell transcriptomes for the 34,736 PBMCs (*n* = 6), grouped by disease (left), and cell types (right). (**C**) Heatmap of all identified clusters after dimensional reduction. The top 10 marker genes colored by their expression level were used for downstream analyses. Clusters were reordered into respective cell types. (**D**) Bar plot showing cell fractions of leukocyte subtypes in patients with gout flare and gout remission, color-coded for the different cell types identified in this study. (**E**) Expression levels of canonical cell markers used to identify cell types. Feature plot represented by color gradient, with low expression depicted by gray and high expression represented by red.

Peripheral blood samples were obtained from the same patients with gout during both flare and remission stages. Then, single cells were isolated and subjected to scRNA-Seq using the 10× Genomics platform (Figure 1A). Following quality control analyses (Supplemental Figure 1, B and C; supplemental material available online with this article; https://doi.org/10.1172/jci.insight.171417DS1), we identified 34,736 high-quality transcriptomes from PBMCs.

We next normalized and clustered the gene expression matrix and conducted dimensionality reduction using t-distributed stochastic neighbor embedding (t-SNE) and graph-based clustering, which resulted in 11 clusters (Figure 1, B and C, and Supplemental Figure 1D). Based on the expression of known marker genes, the 11 clusters could be categorized into 4 known cell lineages: T/NK cells (clusters 1, 3, 4, 5, and 6; *CD3D*, *CD3G*, *NKG7*), myeloid cells (clusters 2, 8, 9, and 10; *HLA*-*DRA*, *CD14*, *FCGR3A*), B cells (cluster 7; *CD79A*, *CD79B*), and 1 unknown cell type (cluster 11) (Figure 1, C and E). Myeloid, B, T, and NK cells constituted a significant portion of the cellular landscape, with no discernible differences in cellular composition between gout flares and remission. These results indicate that the subtype cells might alter their genetic, morphological, and functional levels (Figure 1D). We next proceeded with detailed analyses of the main cell types, to elucidate the differences between the states of gout flare and remission.

### Monocyte subtypes regulate different biological functions during gout flare and remission.

**Figure 2:**
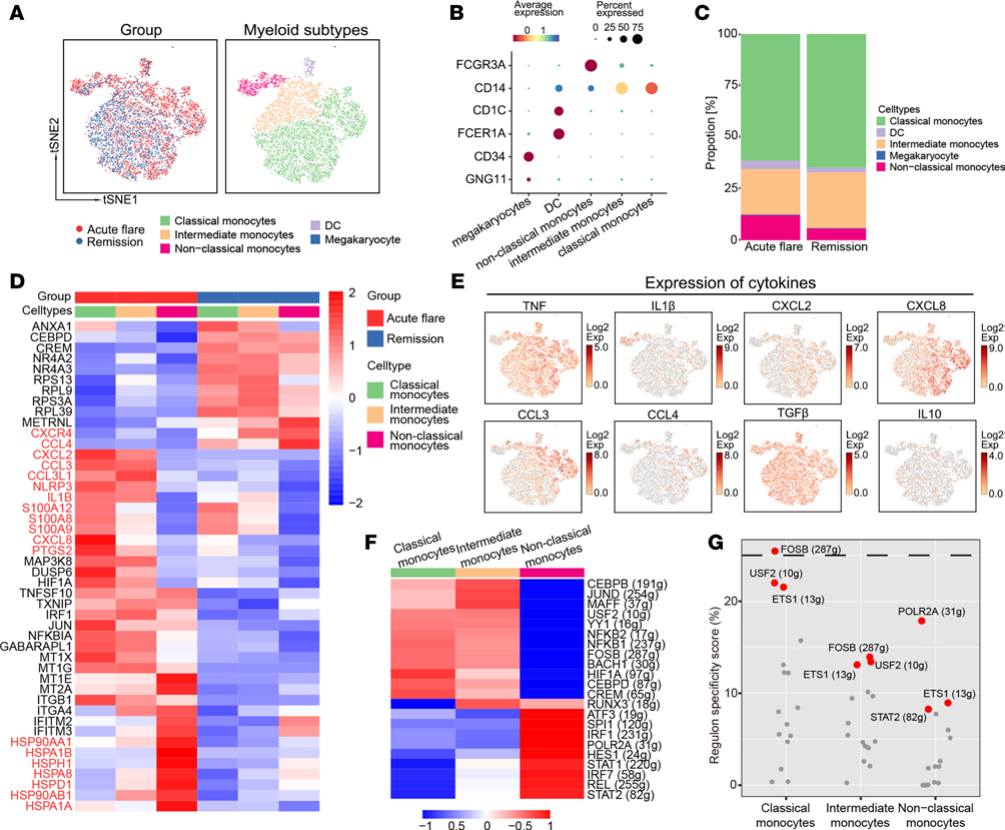
Functions of each monocyte subtype that contribute to immune responses in patients with gout flare and remission. (**A**) The t-SNE representations of integrated single-cell transcriptomes for the 7,499 myeloid cells (*n* = 6), color-coded by disease (left), and cell types (right). (**B**) Expression levels of canonical cell markers used to label clusters. Dot plot represented by color gradient, with low expression depicted by blue and high expression shown in red. (**C**) Bar plot of cell fractions for myeloid cell subtypes stratified by groups. (**D**) Heatmap of the DEGs among monocyte subtypes between gout flare and gout remission. The heatmap is colored by average log(FC). All displayed genes are statistically significant at *P*<0.05. (**E**) t-SNE plots illustrating the expression of characteristic cytokine markers in monocyte subtypes. Feature plot represented by color gradient, with low expression shown in gray and high expression depicted in red. (**F**) Heatmap of the AUC score t values for the expression regulation by transcription factors of monocytes subtypes, as estimated using SCENIC. (**G**) The regulon-specific AUC score t values for the expression regulation by transcription factors of the monocyte subtypes, as estimated by SCENIC.

The 7,499 myeloid cells derived from patients with gout during both the flare and remission stages were divided into 6 clusters and subsequently visualized using t-SNE (Figure 2A). Previous research has shown that monocytes can be subdivided into 3 categories: classical (CD14^+^CD16^−^), intermediate (CD14^+^CD16^+^), and nonclassical (CD14^−^CD16^+^) (11). Of note, classical monocytes (CMs) and nonclassical monocytes (NCMs) have distinct functions in peripheral blood. The CMs are central during the initial inflammatory response, whereas NCMs participate in antiinflammatory functions to maintain vascular homeostasiss (12). Since the majority of myeloid cells in this study were monocytes (96.25%), we focused on monocyte subtypes in gout flares and remission. Lineages of CMs, intermediate monocytes (IMs), NCMs, DCs, and megakaryocytes were identified based on the expression of canonical marker genes (Figure 2B). By calculating the frequency of cell counts contributed by cell subtypes, we identified a higher contribution from NCMs during gout flare (12.14%) than during remission (5.72%). Conversely, the contributions of CMs and IMs were lower during gout flare (61.42% and 21.92%, respectively) as compared with the remission stage (64.71% and 26.91%, respectively) (Figure 2C). Upon comparing the frequency of cell counts contributed by each group, we found that cells from gout flare accounted for 75.32% of NCMs (Supplemental Figure 2A).

Examination of the top differentially expressed genes (DEGs) revealed that CMs and IMs had similar DEG profiles, including cytokine-related (*TNFSF10*, *JUN*, and *NFKBIA*), inflammation-related chemokine (*CXCL2*, *CXCL8*, and *CCL3*), inflammasome-associated genes (*NLRP3* and *IL1B*), and mineral absorption-related genes (*MT2A*, *MT1X*, *MT1E*, and *MT1G*). In contrast, the majority of NCM DEGs was primarily centered around members from the heat shock protein (HSP) family (*HSP90AA1*, *HSP90AB1*, *HSPA1A*, *HSPH1*, and *HSPD1*) (Figure 2D). Similarly, proinflammatory cytokines were upregulated in CMs and IMs during gout flares (Figure 2E and Supplemental Figure 2B). This evidence implies that CMs and IMs, but not NCMs, contribute to the inflammatory response during gout flares.

Utilizing the key motifs identified through SCENIC, we investigated the potential transcription factors (TFs) that could regulate the development of monocyte subtypes. SCENIC revealed a dichotomy between gout flares and remission. The CEBPB, JUND, FOSB, and NFKB1 motifs were highly activated in CMs and IMs, while activation of the REL, IRF1, STAT1, and STAT2 motifs was greatly increased in NCMs (Figure 2F). The regulon-specific scores among monocyte subtypes further indicated that both CMs and IMs have different biological properties from NCMs during gout flares (Figure 2G and Supplemental Figure 2C).

### Augmentation of HLA-DQA1^hi^ NCMs controls the differentiation of monocyte subtypes in gout flare.

**Figure 3:**
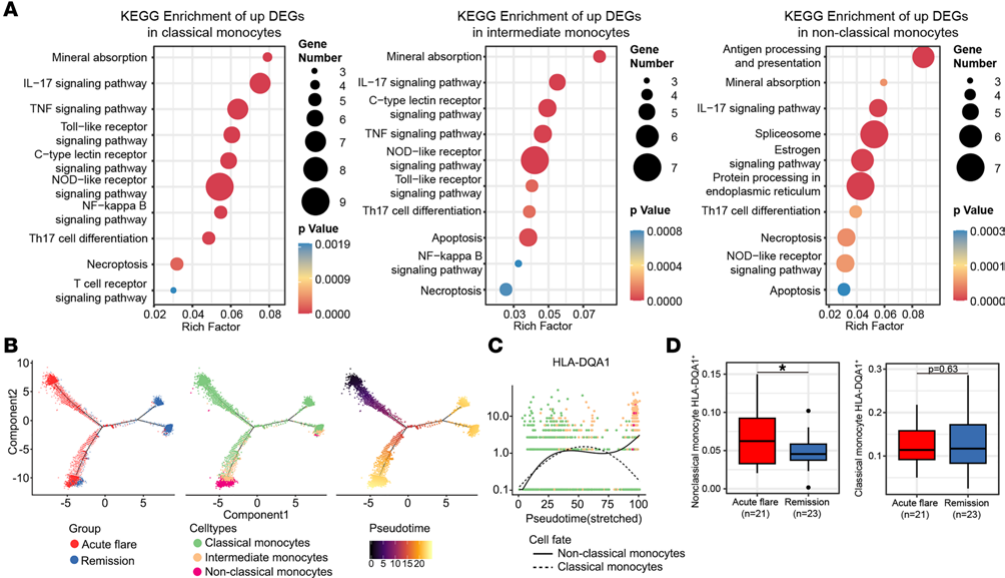
HLA-DQA1^hi^ nonclassical monocytes regulate monocyte subtype differentiation in gout flare. (**A**) KEGG pathway enrichment analysis for classical, intermediate, and nonclassical monocytes (left to right) was performed using the upregulated genes. (**B**) Single-cell trajectory analysis integrating cluster information. (**C**) Dynamics of DEGs (*P*_adj_ of Wilcoxon’s rank-sum test < 0.05, log_2_(FC) > 1) between classical and nonclassical monocytes. (**D**) Representative box plots depicting the expression of HLA-DQA1 in classical and nonclassical monocytes during gout flare and remission. Data presented as median with IQR. The box represent the IQR, which spans from the lower to the upper quartile, while the box whiskers indicate the range of the data, excluding any outliers. Outliers are represented by individual points beyond the whiskers and are defined as values that fall outside the threshold 1.5 times the IQR range. Data from 21 patients with acute gout flare and 23 patients with gout remission are shown. Statistical analysis was performed by 2-tailed Student’s *t* test (*P < 0.05).

The Kyoto Encyclopedia of Genes and Genomes (KEGG) analysis further corroborated our hypothesis regarding the distinct roles of monocyte subtypes. When comparing the upregulated DEGs of CMs and IMs between gout flare and remission, we observed enrichment in pathways including mineral absorption, together with IL-17, TNF, and TLR signaling. In contrast, NCMs were significantly enriched in antigen processing and presentation (Figure 3A).

Pseudotime analyses revealed that 1 branch of CMs progressively differentiated into IMs and then into NCMs (Figure 3B), which is consistent with previous reports (12, 13). The other branches may have distinct biological functions (Figure 3B). Furthermore, this differentiation process was accompanied by the upregulation of HLA family genes, which are integral to antigen processing and presentation (Figure 3, A–C, and Supplemental Figure 2, D and E). Of note, this pattern was not observed in the other branches of CMs, indicating distinct differentiation pathways within the monocyte population.

To validate the observed trajectory trends, we examined an independent cohort for the expression of marker genes associated with monocyte development (Supplemental Figure 3, A–C, and Supplemental Table 1). We found that the expression of HLA-DQA1 increased in NCMs during gout flares but gradually decreased in CMs (Figure 3D). These expression patterns are consistent with those of the 2 trajectories. Collectively, these results demonstrate that an increase in HLA-DQA1^hi^ NCMs is associated with gout flares, possibly through antigen processing and presentation pathways.

### T/NK cell phenotypes in gout flare and remission.

**Figure 4:**
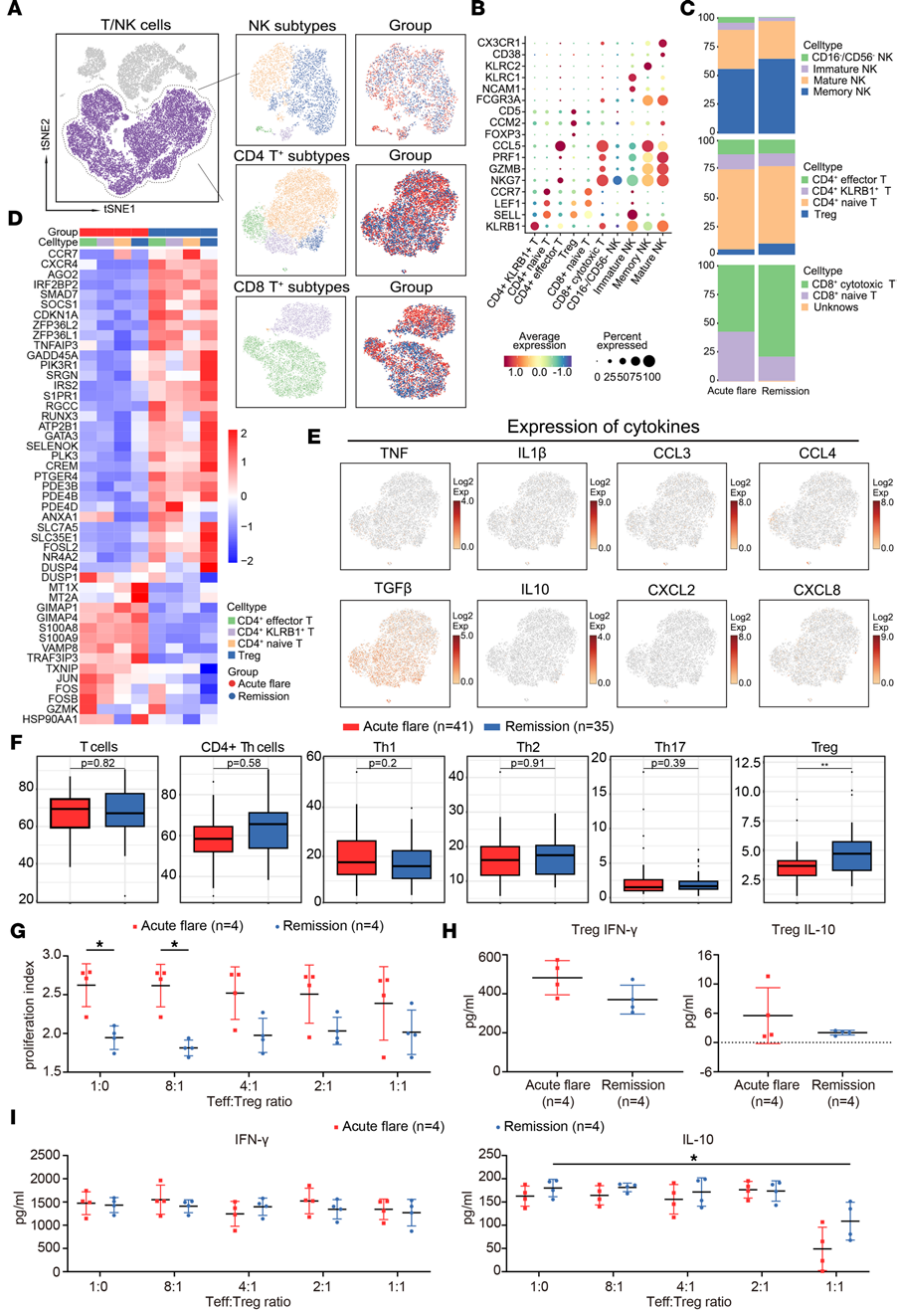
The role of CD4^+^ T cells subtypes in gout flare and remission. (**A**) t-SNE representations of T/NK clusters from gout flare and remission. (**B**) Dot plot for the expression of canonical marker genes in all T cell and NK cell subtypes. (**C**) The proportions of cell subtypes in gout flare and remission. (**D**) Heatmap of DEGs in CD4^+^ T cells based on pairwise comparisons between gout flare and remission. (**E**) The t-SNE plots depict the expression of characteristic cytokines in CD4^+^ T cells. (**F**) The percentage of T, CD4^+^, Treg, Th1, Th2, and Th17 T cells in gout flare (*n* = 41) and remission (*n* = 35). Data presented as median with IQR. The box represent the IQR, while the whiskers indicate the data range without outliers. Outliers are shown as individual points beyond the whiskers and are defined as values outside the 1.5 times the IQR range threshold. Statistical analysis performed by 2-tailed Student’s *t* test (**P < 0.01). (**G**–**I**) Suppressive assay of Tregs (data shown as mean ± SD; *n* = 4 independent experiments). (**G**) The proliferation index by coculture of eFluor 450 dye–labeled autologous Teffs with autologous Tregs isolated from patients with gout flare and those with gout remission. Statistical analysis was performed by 2-way ANOVA (*P < 0.05). (**H**) The production of IFN-γ and IL-10 in cell culture supernatant of Tregs isolated from patients with gout flare and gout remission. Statistical analysis was conducted by 2-tailed Student’s *t* test. (**I**) The production of IFN-γ and IL-10 from the coculture of eFluor 450 dye–labeled autologous Teffs and autologous Tregs, isolated from patients with gout flare and gout remission. Statistical analysis performed by 2-way ANOVA (*P < 0.05).

In our study, 24,923 T/NK cells were detected, which were further clustered into CD4^+^ T cells (TCs) , CD8^+^ TCs, and NK cells (Figure 4A). Based on canonical marker gene expression, we assigned 6 TC subtypes, including CD4^+^ naive TCs, CD4^+^ effector TCs, CD4^+^KLRB1^+^ TCs, Tregs, CD8^+^ naive TCs, CD8^+^ cytotoxic TCs, and 4 NK cell subtypes, including immature, mature, memory, and CD16^–^CD56^–^ NK cells. (Figure 4, B and C).

We subsequently identified DEGs in the CD4^+^ TCs, CD8^+^ TCs, and NK cell subtypes between gout flare and remission (Figure 4D; Supplemental Figure 4B; Supplemental Figure 5, B and C; and Supplemental Figure 6, B and C). We observed significant heterogeneity among NK and CD8^+^ TC types (Supplemental Figure 5, B and C, and Supplemental Figure 6, B and C). The expression of calprotectin (S100A8/A9), and the activator protein 1 family member JUN in both T and NK cells increased during gout flares. The CD4^+^ TCs and CD8^+^ TCs from gout remission were enriched in chemokines, such as CXCR4, as well as various antiinflammatory regulators (TNFAIP3, ZFP36, and SMAD7; an antagonist of TGF-β signaling).

Of note, the expression of most proinflammatory factors in CD4^+^ TCs was almost negligible, with the exception of TGFB1, which was highly expressed. This indicated that CD4^+^ TCs were likely not involved in the inflammatory response to gout (Figure 4E). However, CD8^+^ cytotoxic TCs expressed both proinflammatory CCL4 and antiinflammatory TGFB1 markers (Supplemental Figure 5D). The NK cells expressed CCL4, TGFB1, and the proinflammatory factor CCL3 (Supplemental Figure 6D). Given that TGF-β suppresses the functions of Th1 and Th2 CD4^+^ effector cells, we established an independent cohort to specifically assess the proportion of Th1 and Th2 (Figure 4F and Supplemental Table 2). However, no significant differences were observed between patients with gout flares and those with gout remission. These findings suggest that T and NK cells exhibit less pronounced inflammatory activity than myeloid cells in gout flares.

### The IL-17 signaling pathway triggers adaptive immune responses during gout flares.

KEGG analysis indicated that the DEGs of KLRB1^+^CD4^+^ TCs from gout remission were enriched for the FoxO signaling pathway. In contrast, the DEGs of KLRB1^+^CD4^+^ TCs from the gout flare patient group only exhibited enrichment for the IL-17 signaling pathway (Supplemental Figure 4C). In contrast, in Tregs from gout flare versus remission, the IL-17, NOD-like receptor, and MAPK signaling pathways were enriched, whereas the cGMP-PKG, FoxO, and cAMP signaling pathways were significantly inhibited (Supplemental Figure 4D). We also detected significant upregulation of genes in CD8^+^ cytotoxic TCs, CD8^+^ naive TCs, and NK cells, which were associated with the IL-17 signaling pathway in gout flares (Supplemental Figure 5E and Supplemental Figure 6E). Collectively, these findings support that the IL-17 signaling pathway within the T and NK subtypes elicits robust adaptive immune responses in gout flares.

### The proportion and function of Tregs in gout remission.

Multiple studies have demonstrated that human Th17 cells originate from CD4^+^KLRB1^+^ (CD161^+^) TC precursors (14–16). Our single-cell analysis yielded 11,348 CD4^+^ TCs, which were reclustered into 4 subtypes: naive CD4^+^ TCs, effector CD4^+^ TCs, Tregs, and cells expressing KLRB1 markers (Figure 4B). The percentage of Tregs in patients in remission (5.43%) was higher than that in patients with gout flares (2.57%) (Figure 4C and Supplemental Figure 4A). The balance between Th17 cells and Tregs is critical for maintaining immune homeostasis (10). Furthermore, a decrease in the Treg/Th17 ratio is associated with an inflammatory response during gout flares (17). This indicated that an imbalance between Th17 cells and Tregs is linked to gout development. To determine the changes in TC subtypes in the peripheral blood, we designed an independent cohort to specifically assess alternating TC subtypes (Supplemental Table 2). As expected, only Tregs were significantly increased during gout remission, which was consistent with our scRNA-Seq results (Figure 4F and Supplemental Figure 4, E and F).

We next classified Tregs into 4 clusters and visualized these using t-SNE to investigate their functions (Supplemental Figure 7A). A heatmap of the top DEGs in each cluster revealed that clusters 1 and 2 shared similar functional characteristics, marked by the high expression of *IL2RA*, *FOXP3*, cytotoxic T-lymphocyte–associated protein 4 (*CTLA4*), *TIGIT*, and *CCL4* (Supplemental Figure 7, B and C). Interestingly, we found that inhibitory genes, such as *CTLA4* and inducible costimulator (*ICOS*), were significantly upregulated during gout remission (Supplemental Figure 7D). Several studies have demonstrated that ICOS facilitates the production of antiinflammatory factors, whereas CTLA4 helps to maintain the suppressive activity of Tregs (18, 19). Furthermore, cluster 3 exhibited high expression of *ANXA1*, which inhibits phospholipase A2 and has been associated with antiinflammatory activity in gout (20, 21) (Supplemental Figure 7, B and C). These findings led us to posit that the role of Tregs in gout remission is likely related to their suppressive and antiinflammatory activities.

To validate the function of Tregs in vitro, we performed a suppression assay by coculturing eFluor 450 dye–labeled autologous effector TCs (Teffs) with autologous Tregs for 4 days in the presence of anti-CD3 and anti-CD28. When the ratio of Teffs/Tregs was either 1:0 or 8:1, Tregs isolated from patients in gout remission demonstrated significantly enhanced suppressive capabilities compared with those isolated from patients with gout flare (Figure 4G). However, as the concentration of Tregs in the coculture system increased, the suppressive activity of Tregs during gout flares and remission did not change significantly (Figure 4G).

To further investigate the secretion of cytokines under various Teff/Treg ratios, we found that both the proinflammatory cytokine IFN-γ and the antiinflammatory cytokine IL-10 exhibited higher concentrations in the culture supernatant during gout flare compared with gout remission (Figure 4, H and I). Interestingly, since the concentration of Tregs increased in the coculture system, the levels of IFN-γ exhibited minimal discernible variation. Conversely, the secretion of IL-10 decreased as the concentration of Tregs increased, particularly when the ratio of Teffs/Tregs was 1:1 (Figure 4I). As such, we postulated that an increase in Tregs during gout remission may enhance the suppressive function of Tregs, which is potentially mediated by CTLA4 and ICOS.

### Major alterations of the intercellular signaling network between gout flare and remission.

**Figure 5:**
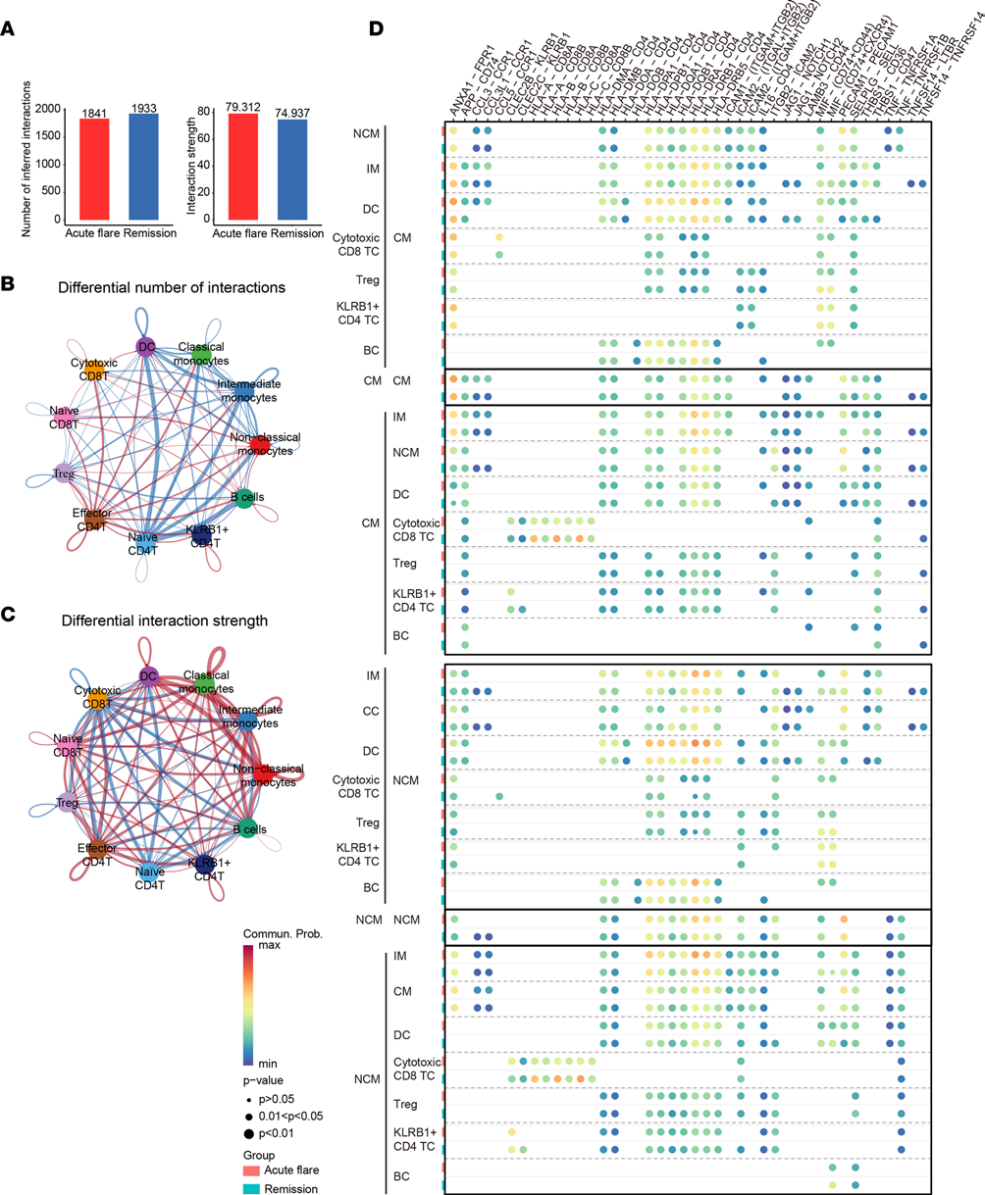
CellChat analysis highlighting the intercellular communication between acute gout flare and remission. (**A**) Comparison of the inferred interaction number (left) and interaction strength (right) in gout flare and remission. (**B** and **C**) Dot plots of the inferred interaction number (**B**) and interaction strength (**C**) between acute gout flare and remission. Blue lines indicate that the displayed communication is increased in gout remission, whereas red lines indicate its increase during gout flare. (**D**) Comparison of the significant ligand/receptor pairs between gout flare and remission, which contribute to signaling from classical monocytes (CM) and nonclassical monocytes (NCM) to DCs, intermediate monocytes (IM), and T cells (TC), including Treg, KLRB1^+^CD4^+^ TC, cytotoxic CD8^+^ TC, and B cells (BC). Dot color reflects communication probabilities, and dot size represents the computed *P* values. The empty space indicates a communication probability of zero. The *P* values were calculated based on 1-sided permutation test.

We used CellChat to quantify and visualize intercellular communication among various immune cell subtypes. In general, the number of inferred interactions was higher during gout remission than that during gout flares. However, the strength of these interactions is typically enhanced during gout flares (Figure 5A). To further delineate the interactions between cell types across the 2 stages of gout, we used circular plots to visualize the interaction quantity. The number of inferred interactions between naive CD4^+^ TCs and other myeloid cell subtypes increased during gout remission (Figure 5B). Conversely, the strength of cell communication between monocyte subtypes and DCs was enhanced during gout flares (Figure 5C). By comparing the information flow between gout flares and remission, we identified that 15 of 42 pathways were highly active during gout flares, including 9 pathways involved in inflammatory and immune responses, such as CCL, IL-16, MHC-II, CLEC, GLAECTIN, ITGB2, and ALCAM (Supplemental Figure 8A). There were only 16 pathways active in gout remission, including inflammatory and coagulation-related pathways LIGHT, TWEAK, NOTCH, COLLAGEN, THBS, MIF, and TGF-β. These results strongly suggest that monocyte subtypes play crucial roles in regulating a variety of immune cell types.

Chemokines and cytokines promote global inflammatory responses during gout flares. We observed that the CCL and IL-16 signaling pathways among monocyte subtypes were greatly increased in gout flares (Supplemental Figure 8, B and C). Furthermore, the CCL5 ligand and its receptor, CCR1, acted as major signals from cytotoxic CD8^+^ TCs to CMs primarily during gout flares compared with during remission (Figure 5D). However, this ligand/receptor pair contributed to gout remission from cytotoxic CD8^+^ TC to NCMs. The IL-16 ligand and its CD4 receptor were also highly active during gout flares in CMs, myeloid cells, Tregs, and KLRB1^+^CD4^+^ TCs (Figure 5D). Compared with remission, we observed that both ITGB2 and ICAM signaling were increased in gout flares, suggesting an important role for cell adhesion signaling (Supplemental Figure 8, D and E). In addition to a slight increase in the number of interactions involving MHC-II signaling during gout flares, we observed a distinct activation of the ligand HLA-DQA1 and its cognate receptor CD4. Specifically, this activation was observed in NCMs communicating with other cell types during gout flares. This finding is consistent with our previous results from the trajectory analysis of monocyte subtypes (Figure 5D and Supplemental Figure 8F).

In contrast, we discovered that the THBS, NOTCH, and ICOS signaling pathways were downregulated during gout flares (Supplemental Figure 8, G–I). Compared with NCM, both THBS and NOTCH signaling were highly activated in CMs, with a greater interaction strength and more signaling targets.

Taken together, CellChat analysis revealed differential activation of signaling pathways between CMs and NCMs during gout flares and remission.

### Monocyte subtypes act as primary peripheral contributors of the inflammatory response during gout flare.

Our analysis of gene list scores revealed that myeloid cells had higher inflammatory and cytokine scores than the other cell types (Supplemental Table 3). This analysis indicated that myeloid cells had higher inflammatory and cytokine scores than other cells (Supplemental Figure 9, A and B). Furthermore, monocytes, particularly CMs, showed significantly elevated inflammatory scores (Supplemental Figure 9, C and D). Based on these findings, we investigated the differences in the inflammatory signatures of each cell subtype between gout flares and remission. Of note, both CMs and IMs demonstrated a robust inflammatory response during gout flares, consistent with our monocyte findings (Supplemental Figure 9E). However, subtypes of T and B cells exhibited higher inflammatory scores during gout remission, despite rising cytokine scores during gout flare (Supplemental Figure 9E). These observations suggest that CMs may be the principal contributors to the inflammatory responses during gout flares.

### Single-cell arachidonic acid (AA) metabolic activity in gout flare and remission.

**Figure 6:**
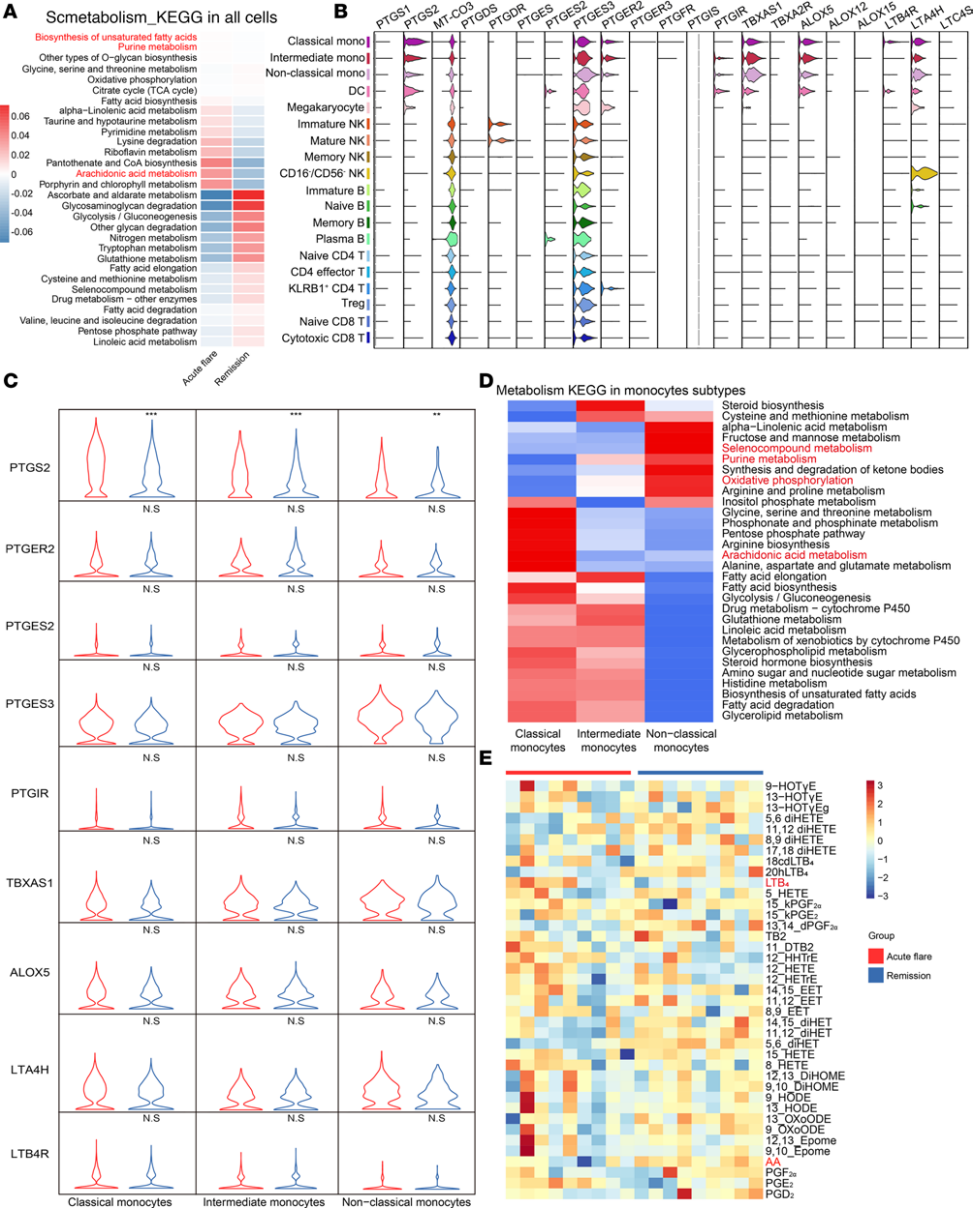
Metabolic properties of monocyte subtypes between acute gout flare and remission. (**A**) The heatmap of significantly altered metabolic pathways for all PBMCs between gout flare and remission. (**B**) Violin plots of selected marker genes (upper row) related to the arachidonic acid pathway for multiple cell subpopulations. The left column presents the cell subtypes identified based on combinations of marker genes. (**C**) Violin plots for the average expression of genes related to the arachidonic acid pathway in each monocyte subtype between acute gout flare and remission. The *P* values were calculated using a 2-sided Wilcoxon rank-sum tests. Data are from single-cell transcriptomes of 3 independent patients with gout (**P<0.01, ***P<0.001). (**D** and **E**) Heatmaps of the significantly altered metabolic pathways in monocyte subtypes (**D**) and all PBMCs (**E**) between gout flare and remission.

To understand the metabolic characteristics of gout flares and remission at single-cell resolution, we used a new R package, scMetabolism, to quantify metabolic activity. Following this approach, the AA pathway emerged as one of the most active metabolic pathways during gout flares (Figure 6A). Conversely, ascorbate and aldarate metabolism as well as glycosaminoglycan degradation were highly enriched during gout remission. Myeloid cells, particularly monocytes, had higher AA scores during gout flares than during gout remission. (Supplemental Figure 10, A and B). The prostaglandin-endoperoxide synthase 2 (PTGS2), PTGER2, TBXAS1, ALOX5, and LTA4H proteins were highly expressed in myeloid cell subtypes (Figure 6B). Notably, the levels of PTGS2 (also known as cyclooxygenase 2; COX2), a key enzyme in prostaglandin biosynthesis, were significantly higher in gout flares among CMs, IMs, and NCMs (Figure 6C). Furthermore, we calculated metabolic activity across monocyte subtypes. The AA metabolism exhibited a strong correlation with CMs and IMs but was weakly associated with NCMs, in line with our previous observations regarding the characteristics of monocyte subtypes (Figure 6D). Oxidative phosphorylation, selenocompound metabolism, and purine metabolism were enriched in the NCMs (Figure 6D).

Recognizing the critical role of the AA pathway in gout flares, we quantified AA metabolites from plasma samples of an independent paired cohort using liquid chromatography–mass spectrometry (LC-MS) (Supplemental Table 4). A heatmap of the metabolic profiles is shown in Figure 6E. A 2-dimensional scatter plot for orthogonal partial least squares–discriminant analysis (OPLS-DA) visually separated gout flares from gout remission (Supplemental Figure 10C). The S plot and corresponding VIP values showed that AA, 5,6-dihydroxyeicosatrienoic acid (diHET); 5,6-dihydroxy-8Z,11Z,14Z,17Z-eicosatetraenoic acid (5,6-DiHETE); 9-oxo-octadecadienoic acid (9-oxoODE); leukotriene B4 (LTB4); and 20hLTB4 were the primary contributors for differentially identifying gout flares and remission (Supplemental Figure 10D). The plasma levels of AA, diHET, 5,6-DiHETE, 9-oxoODE, and 20hLTB4 significantly decreased during gout flares (Supplemental Figure 10E). However, LTB4 was significantly increased in gout flares, consistent with the results of our previously published study (22) (Supplemental Figure 10E). Collectively, these data underscore the pivotal role of AA metabolism during gout flares and highlight that monocyte subtypes with high PTGS2 expression are critical during gout flares.

## Discussion

Several recent studies have examined single-cell–level differences in peripheral blood immune cells between patients with gout flares and healthy individuals (23, 24). However, our work focused on investigating the gradual shift in the landscape of such cells during the transitional stages from gout flares to remission, which we believe offers valuable insights with considerable clinical therapeutic potential.

Hyperplasia of the synovial membrane and neutrophil infiltration, accompanied by monocytes and macrophages, represent typical clinicopathological hallmarks of gout flares in localized joints (1, 2). Rapid IL-1β production by activation of the NLRP3 inflammasome is the characteristic mechanism of the gout inflammatory response (25). Parallel to the localized joint gout flare, our current work revealed that circulating CMs, IMs, and DCs were highly enriched in proinflammatory factors, such as NLRP3, IL-1B, and TNF, which exhibited relatively low expression in blood NCMs and other PBMCs (Figure 2E and Supplemental Figure 2B). We observed that the upregulated genes within the CMs and IMs were predominantly related to the TNF and TLR signaling pathways (Figure 3A). This evidence suggests that circulating CMs and IMs are also the principal sources of the inflammatory response in gout flares, potentially through the TLR and TNF signaling pathways.

The HLA-DQA1 is widely recognized for its role in influencing the risk of various autoimmune diseases, including vitiligo, celiac disease, idiopathic membranous nephropathy, and multiple sclerosis (26–30). Interestingly, our results demonstrate the upregulation of HLA-DQA1 during the differentiation of CMs to NCMs, concomitant with the activation of antigen presentation pathways. We observed a significant increase in HLA-DQA1^hi^ NCM expression during gout flares (Figure 3D). Although gout is primarily driven by the deposition of urate crystals and the subsequent immune response, the specific antigens responsible for triggering and sustaining inflammation in gout remain under investigation. Based on our findings, we propose that the upregulation of HLA-DQA1 in NCMs with antigen presentation during gout flares is not specific to urate crystals; rather, it is implicated in damage-associated molecular patterns produced by the inflammation associated with gout flare (31). In contrast, the upregulation of circulating NCMs during gout flares may reflect their mobilization from the BM or other tissue reservoirs, potentially as a compensatory response to the inflammatory process. Therefore, the high expression of HLA-DQA1 in NCMs with antigen processing and presentation may be an underlying immune mechanism during gout flares.

Here, we sought to elucidate the role of circulating TC subtypes in the initiation, progression, and resolution of gouty arthritis. Our single-cell results and flow cytometric analysis confirmed an increase in Tregs during gout remission. The Tregs exhibit significantly enhanced suppressive capabilities during remission compared with those observed during gout flares. In a coculture system with escalating concentrations of Tregs, the suppressive activity of Tregs was not significantly different between gout flares and remission. However, the secretion of the antiinflammatory cytokine IL-10 was markedly decreased. This suggests that the suppressive function of Tregs can be modulated within an optimal concentration range (32). Moreover, healthy individuals had a higher number of Tregs than patients in gout remission (Supplemental Figure 11). This observation led us to hypothesize that Tregs are depleted during the inflammatory process, which may offer valuable insights into the functional dynamics of Tregs and have significant implications for cellular therapy in the management of gout.

Previous research has shown that the expression of CD80/86 in antigen-presenting cells (APCs) is downregulated by the Treg surface membrane protein CTLA-4, following activation of the TC receptor by the MHC on APCs (33). In this study, we also observed that Tregs produced during gout remission highly expressed CTLA4 and ICOS, which further prompted us to examine the interaction between Tregs and APCs (Supplemental Figure 7D). Our results reveal that all APCs, except NCMs, had different numbers of interactions during gout flares (Figure 5B). In terms of the strength of such differential interactions, all APCs, except NCMs, received incoming signals from Tregs during gout remission (Figure 5C). Tregs interacted with a larger number of ICOS signaling networks during remission, and HLA-DQA1 interacted predominantly with NCMs rather than with CMs (Figure 5D and Supplemental Figure 8I). These findings underscore the potential of Tregs and HLA-DQA1^+^ NCMs in the peripheral blood as targets during the transition between gout flare and remission.

We also conducted a comprehensive analysis of both the immune cell and systemic inflammatory responses. The COX2 selective inhibitors can swiftly and effectively alleviate pain during gout flare (34). We identified numerous metabolic processes, including AA metabolism, which were significantly increased during gout flares. Correspondingly, genes related to AA metabolism were highly expressed in the myeloid subtypes, particularly in CMs and IMs (Figure 6, A and B). More importantly, high expression of PTGS2 in monocyte subtypes was detected in patients with gout flares (Figure 6C), consistent with the MSU-induced PTGS2 expression in human monocytes (35). To validate these findings, we used LC-MS to quantify the metabolites derived from multiple polyunsaturated fatty acids. We observed that the level of AA greatly increased during gout remission, in agreement with previous studies (36). The *PTGS2* is a key gene that converts AA into prostaglandins G2 and H2 (37). Our results demonstrate the upregulation of PTGS2 in monocyte subtypes that are involved in gout flares and remission. Therefore, the elevated expression of PTGS2 in circulating monocyte subtypes plays a pivotal role in gout flares and could underpin the molecular basis for the use of COX2 selective inhibitors as a first-line treatment for gout flares.

The LTB4 is a metabolite of AA and is a potent mediator of gout flares (38). Previous studies demonstrate that LTB4 is produced by human peripheral polymorphonuclear leukocytes exposed to MSU (39). In our work, blood LTB4 concentration increased during gout flares, which further supports the involvement of AA metabolites from PBMCs in gout flares and remission (22). Despite the 5-lipoxygenase (5-LOX) enzyme mediating the biosynthesis of LTB4, we found no difference in the expression of arachidonate 5-LOX (*ALOX5*), the gene encoding 5-LOX, between gout flare and remission. This suggests that the increased level of LTB4 in the blood during gout flares may not be directly related to PBMC activity.

We analyzed PBMCs from the same patient during gout flare and remission using scRNA-Seq, which largely assisted in the avoidance of the variation caused by genetic background. However, our study had certain limitations. First, owing to technical constraints, we were unable to analyze neutrophils within PBMCs. Second, we focused on PBMCs, which may not fully represent the local inflammatory and immune responses that occur during gout flares and remission. Third, we cannot rule out the possibility that antiinflammatory medications previously used to control gout flares and other drugs may have influenced the scRNA-Seq findings. Finally, to confirm the putative roles of monocyte subtypes and Tregs based on transcriptomics data, additional protein analyses and functional validation are required.

In summary, here we characterized marker changes in the immune cell composition and inflammatory features of human PBMCs at the single-cell level throughout the transitional stage from gout flare to remission. During gout flares, CMs and IMs exhibit proinflammatory functions, HLA-DQA1^hi^ NCMs are involved in antigen processing and presentation, and PTGS2^hi^ monocyte subtypes enhance AA metabolism activity. Interestingly, abnormal Treg responses were detected in patients in gout remission. Our study provides promising insights regarding the monocyte subtypes and Tregs during gout flares and remission, which may serve as potential targets for investigating recurrent gout attacks and disease progression.

## Methods

### Patients.

This study was approved by the Medical Ethics Committee of the Shanghai Tenth People’s Hospital of Tongji University (SHSY-IEC-4.1/21-246/01). Written informed consent was obtained from all male participants aged 18 years or older. The participants in the gout flare group had an onset of acute gout flares within 24 hours, meeting the 2015 ACR/EULAR classification criteria (40). Participants in the gout remission group were in the intercritical phase, which was defined as the remission of gout flare and normal erythrocyte sedimentation rate or C-reactive protein level over the last 3 months (the assessment periods were similar for all patients) (41). The MSU crystals were detected in all recruited patients using B-mode ultrasonography or dual-source dual-energy CT (Supplemental Figure 1A). All patients underwent treatment with COX2 inhibitors (administered for 3–5 days) prior to blood sampling during a gout flare. Once patients entered a state of gout remission, the treatment regimen remained unchanged from that before the gout flare. The 2 groups were fully age matched. The exclusion criteria were corticosteroid or indomethacin use in the past 3 months, septic arthritis or other joint diseases (such as rheumatoid arthritis), history of diabetes, renal dysfunction (estimated glomerular filtration rate < 30 mL/min/1.73 m^2^), and other conditions that may increase the level of blood uric acid. All eligible patients were subjected to scRNA-Seq analysis, flow cytometry validation of monocyte and TC subtype results, and LC-MS/MS validation of AA metabolites (Supplemental Tables 5–7).

### Cell isolation.

A 2 mL sample volume of peripheral blood was obtained from patients with gout in EDTA-anticoagulated medium, and whole blood was diluted with 1× PBS. Equal volumes of Ficoll lymphocyte separation solution (Solarbio, P4350) were added to a 50 mL centrifuge tube, and the diluted blood was slowly spread onto the lymphocyte separation solution. Centrifugation was conducted at 20°C with a horizontal rotor at 450*g* for 20 minutes, and brake was set to 0. The intermediate white membrane cells were collected into a 15 mL centrifuge tube. The white membrane layer was washed with 10 mL of 1× PBS and centrifuged at 300*g* for 10 minutes at room temperature. After discarding the supernatant, the cells were resuspended in 5 mL 1× PBS, centrifuged at 300*g* for 10 minutes at room temperature, and washed twice. The supernatant was discarded, and the cells were resuspended in 1 mL RPMI 1640 medium (Corning, 10-040-CVR) supplemented with 0.04% BSA. Cell viability and concentration of single-cell suspensions were determined using a Luna cell counter or trypan blue staining.

### scRNA-Seq data preprocessing.

All scRNA-Seq downstream libraries were generated with Chromium Single cell 3′ Reagent v2 kits, following the manufacturer’s protocol (10× Genomics). The PBMC suspensions were partitioned with oil, reverse transcription reagents, and unique 10× barcode gel beads to generate single-cell gel beads in emulsions (GEMs). Following the generation of GEMs, the barcoded full-length cDNA was purified and amplified by PCR. Then, libraries were prepared for sequencing using Illumina Novaseq 6000, and 150 bp paired-end reads were generated.

Raw sequencing data of patients were processed to demultiplex cellular barcodes using Cell Ranger v.3.1.0. Sequencing reads were aligned to the hg38 human reference genome, and a unique molecular identifier (UMI) was assigned using STAR (42). Subsequently, the gene expression matrices of each patient were obtained and further analyzed using Seurat v.3.2.3. To filter low-quality cells and potential multiplet captures, we removed cells using the following criteria: (a) total UMI counts < 1,000; (b) gene numbers < 300 or > 7,000; and (c) percentage of mitochondrial genes > 10%. The function “DoubletFinder” was used to detect and filter doublets. After quality control testing, 34,736 single cells and 11,506 genes were included for normalization, scaling, and integration with default parameters. Using the “FindVariableFeatures” function, we selected 4,000 highly variable genes from the data sets for downstream analysis (43). To remove batch effects in the scRNA-Seq data, mutual nearest neighbor analysis was performed using the R package batchelor (44). After centering and scaling the top variable genes, principal component (PC) analysis was performed to reduce dimensionality. We computed a k-nearest neighbor graph with Euclidean distances based on the space of the first 30 significant PCs. Finally, t-SNE was performed to realize unsupervised clustering, which was visualized on a 2-dimensional plot.

### Identification of marker genes and cell type annotation.

To identify marker genes, DEG analysis among clusters was performed through the Seurat function “FindAllMarkers” or “FindMarkers” with default parameters. *P* < 0.05 and |fold change (FC)| > 1.5, were used as the criteria to select DEGs with significant alteration in their expression levels. Based on the expression of canonical marker genes, the 11 clusters were assigned to 4 major cell types: myeloid cells (HLA-DRA), TCs (CD3D, CD3G), NK cells (CCL5, NKG7, GZMA), B cells (CD79A, CD79B), and 1 unknown cell type. In a small number of cases, clustering was insufficient to separate cytotoxic TCs from NK cells. Therefore, we further isolated each major subtype and reprocessed them using the same normalization and integration approach as previously described.

### DEGs and pathway enrichment analyses.

To determine the differentiation of the transcriptional patterns between gout flare and remission, the DEGs between the 2 groups obtained for each cell type were analyzed using the Wilcoxon rank-sum test (*P* < 0.05, |FC| > 0.58). The DEG pathways between the 2 groups were processed using KEGG pathway enrichment analysis, based on hypergeometric distribution (45). *P* <0.05 was chosen as the cutoff significance threshold.

### Motif enrichment and regulatory network.

The SCENIC19 v.1.1.2 pipeline was run as previously described to perform TF enrichment analysis of monocyte subtypes based on the motif database for RcisTarget, GRNboost, and AUC (46).

We calculated the regulon specificity score to assess the cell type specificity of each predicted regulon (47). Specifically, the Jensen-Shannon divergence was calculated between each vector of binary regulon activity overlapping with the type of cell assignment. The connection specificity index for all regulons was measured using the scFunctions package.

### Pseudotime analysis.

To construct the monocyte trajectory, the Monocle2 algorithm was used to order monocytes and reduce their dimensionality (48). Monocyte gene matrix counts were converted into CellDataSet objects using the ImportCDS function. Only genes with mean expression ≥ 0.1 were used in the trajectory analysis. With the differentialGeneTest function, ordered genes (*q* < 0.01) were selected to likely contribute to cell-to-cell ordering along the pseudotime trajectory. A dimensionality reduction cluster analysis and trajectory inference were subsequently performed. We displayed the changes in marker genes within monocyte subtypes in the differentiation trajectory based on the plot_genes_in_ pseudo-time function. The branched expression analysis modeling (BEAM) function was then applied to detect marker genes that separated the cells into the identified states. A clustered heatmap of the expression patterns of the hub genes in branches 1 and 2 was generated using Monocle’s heatmap visualization tool.

### Flow cytometry.

After blocking with Fc, the PBMCs were labeled with membrane fluorescent antibodies for 30 minutes on ice in the dark and washed with PBS containing 2% BSA (Sigma-Aldrich) according to the manufacturer’s instructions. After washing, the samples were immediately analyzed using BD FACSverse. To analyze primary human monocyte subtypes, Tregs and Th17 cells were stained with specific antibodies, as listed in Supplemental Tables 8 and 9.

### Treg suppressive assay.

PBMCs were isolated from gout flare and gout remission peripheral blood samples using density gradient centrifugation (450*g* for 20 minutes at 20°C). The CD4^+^CD25^+^CD127^lo/−^ Tregs and CD4^+^CD25^–^ Teffs were then obtained from autologous PBMCs using magnetic bead separation (catalog 18063; Stemcell Technologies). The Tregs were cocultured with eFluor 450–labeled Teffs at different Treg/Teff ratios in the presence of anti-CD3/CD28 beads (catalog 10971; Stemcell Technologies). After 4 days of coculture, the proliferation of Teffs was assessed by flow cytometry based on the eFlour 450 dilution (catalog 00-422; eBioscience). The suppressive function of Tregs was determined by calculating the proliferation index of Teffs using the FlowJo software (v10.8.1).

### ELISA for IFN-γ and IL-10.

To evaluate the suppressive capacity of Tregs, functional assays, such as cytokine production assays, were conducted by ELISA. Cell culture supernatant from coculture of Teffs and Tregs was used to measure IFN-γ and IL-10 using MultiSciences ELISA kit (LiankeBio), following the manufacturer’s protocol.

### Ligand/receptor interaction analysis by CellChat.

To understand the potential cellular interactions between monocyte subtypes and other cell subtypes, including CD4^+^KLRB1^+^ TCs, CD8^+^ cytotoxic TCs, and B cells from gout flare and remission, CellChat (v 1.1.3) based on the CellChatDB database of ligand/receptor pairs in humans was applied to our scRNA-Seq profiles, so as to construct a cell-to-cell communication network. First, we provided the normalized expression matrix as input to produce the CellChat object using the createCellChat function. Second, the data were preprocessed with the identifyOverExpressedGenes, identifyOverExpressedInteractions, and ProjectData functions using default parameters. Then, the potential ligand/receptor interactions were calculated with the function of computeCommunProb, filterCommunication (min.cells = 10), and computeCommunProbPathway. Finally, the collective cell communication network was compiled and visualized by circle and bubble plots. The information flow for each signaling pathway was calculated and compared between gout flares and remission. Intercellular communication between gout flares and remission was analyzed and compared with explore changes in specific signaling pathways.

### Gene signature score evaluation.

Inflammatory, cytokine, and AA scores were assessed using the AddModuleScore function with default parameters in the Seurat R package (49). We curated a gene set of inflammatory and cytokine from the reported references (50) and a gene set termed KEGG_ARACHIDONIC_ACID_METABOLISM from MSigDB (http://www.gsea-msigdb.org/gsea/msigdb). To confirm the most promising cell types associated with gout flares and remission, we performed a 2-tailed *t* test for each subtype score.

### scMetabolism.

The scMetabolism is a package that quantifies metabolic activity at the single-cell level and has broad applications in scRNA-Seq data sets (51). First, scMetabolism requires imputation of a Seurat object containing the UMI count matrix. Second, we generated a list of high-quality metabolic sets based on reported gene sets or the KEGG database to quantify the metabolic pathway activity using VISION. Finally, single-cell metabolic activity was visualized and compared using a heatmap.

### Metabolic profiling analysis.

Lipid mediator standards, including calibration analytes and internal standards, were purchased from GlpBio. Methanol (MeOH, catalog A456), acetonitrile (ACN, catalog A955), water (catalog W6), and 2-mL vials (catalog C4000-2W) with caps were purchased from a Thermo Fisher Scientific.

Plasma samples were prepared for LC-MS/MS analysis as follows. First, 500 μL plasma was mixed with 100 μL formaldehyde and vortexed for 5 minutes. Then, 1 mL ethyl acetate (EA, Shanghai Yuanye) was added to the sample and vortexed for 10 minutes at 25°C. The mixture was centrifuged at 14,100*g* for 10 minutes at 4°C; subsequently, the entire supernatant was collected into a 1.5 mL centrifuge tube for later use. Next, 1.5 mL EA was added to the precipitate, and the mixture was centrifuged at 16,200*g* for 10 minutes at 4°C. The supernatant was then collected into a 1.5 mL centrifuge tube. This step was repeated for an additional 2 times. The dried sample was resuspended in 120 μL formaldehyde (containing 10 ng/mL internal standard) and vortexed for 5 minutes. Finally, after centrifugation at 14,100*g* for 10 minutes at 4°C, the 6 μL supernatant was used for LC-MS/MS analysis.

Oxylipins in the supernatant were separated on an LC gradient for 10 minutes using an analytical column (Acquity UPLC BEH C18 [1.7 μm, 2.1 mm × 100 mm]). Briefly, the sample was loaded onto the column in buffer A (0.1% v/v formic acid) and eluted at a flow rate of 300 μL/min and a temperature of 40°C over 10 minutes in a gradient ranging from 5% to 90% of buffer B (0.1% v/v formic acid, 100% v/v ACN). The gradient program was optimized to successfully separate the compounds in the same multiple reaction monitoring mode and achieve maximum intensity of most analytes. Samples were injected into an AB SCIEX 5500 Qtrap mass spectrometer for further analysis as previously described (52).

### Statistics.

Statistical analysis was performed and plots were generated using GraphPad Prism 9(GraphPad Software Inc.) and IBM SPSS Statistics 27 (IBM Corp.). The Kolmogorov-Smirnov test was used to determine the data distribution. Among the continuous data, variables with normal distribution are depicted as mean ± SD, while those with skewed distribution are shown as median ± interquartile range (IQR). Categorical variables are presented as percentages. *P* <0.05 was considered statistically significant in the 2-tailed Student’s *t* test, nonparametric test, or 2-way ANOVA. Student’s *t* test was used to assess the significance of differences in TF expression between samples from gout flare and remission. Two-way ANOVA was used for the Treg suppressive assay. The OPLS-DA and S-plot analyses were conducted using SIMCA 13 (Umetrics).

### Data availability.

The scRNA-Seq data have been deposited in the GEO database (GSE211783). Values for all data points in graphs are reported in the Supporting Data Values file.

## Author contributions

Hanjie Yu and WX supervised the study and drafted the manuscript. YS, XL, SW, HB, HG, GC, JC, DH, LQ, YT, and Liya Wang supervised sample collection and clinical annotation, with important assistance by ZA, who performed data analysis, and with significant contributions from DZ and Hanqing Yu, who conducted the flow cytometry analyses. AP designed the study, and Ling Wang contributed to critical data interpretation. The authorship order among co–first authors are as follows: Hanjie Yu (first) and WX (second) based on their contributions of drafted the manuscript. All authors have read and provided comments on the manuscript.

## Supplementary Material

Supplemental data

Supplemental tables 1-8

Supporting data values

## Version 1 12/08/2023

Electronic publication

## Version 2 12/18/2023

Anonymized patient identification numbers were added to the supplemental material
