# Supplementary material for: Single-cell transcriptomics reveals variations in monocytes and Tregs between gout flare and remission
Source: JCI Insight. 2023 Dec 8;8(23):e171417. doi: 10.1172/jci.insight.171417 (PMC10795830; doi:10.1172/jci.insight.171417)
Supplement: Supplemental data [file jciinsight-8-171417-s203.pdf]

A

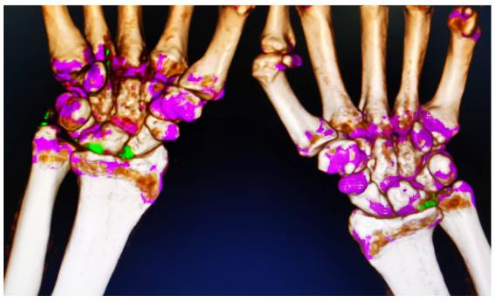

B

Batch name	Capture cell	Median genes per cell	Median UMIs per cell	Cell passed QC
A1	6446	1706	4948	3631
A2	6840	1811	5808	4405
A3	15110	1229	4472	11632
B1	6019	1656	6030	5305
B2	5005	1728	6265	4411
B3	15792	1412	4644	5352

C

D

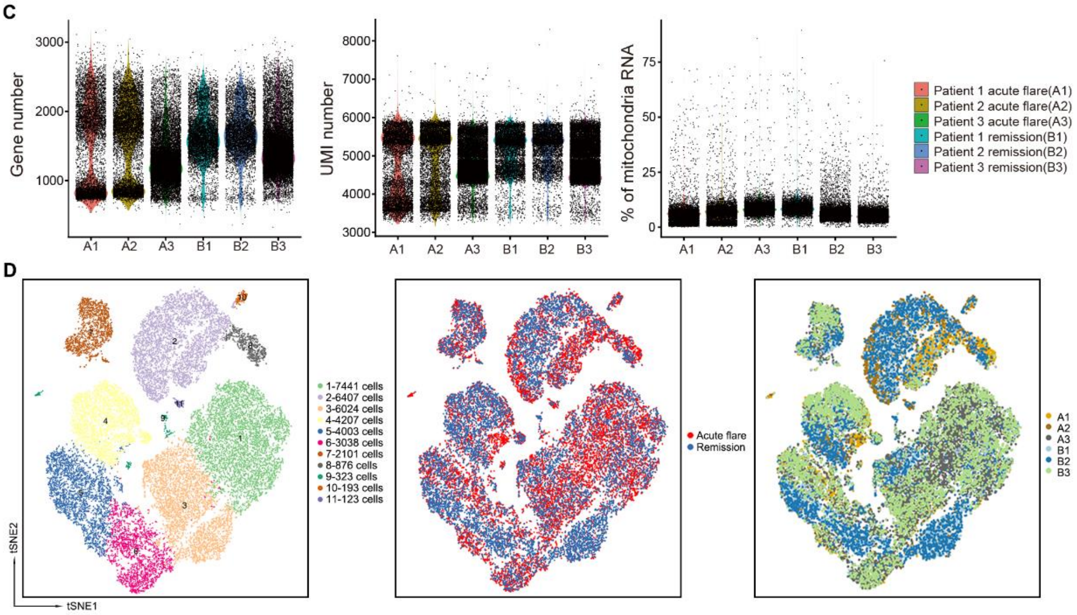

Supplemental Figure 1 Quality control of single-cell data for PBMC samples from gout patients. (**A**) Dual energy CT picture of gout patient. (**B**) Summary of captured cells, median genes per cell, median UMIs per cell, and the number of cells that passed quality control (QC) in distinct batches of single-cell data from gout patients. (**C**) Box plots showing the gene number (top panel), UMI number (bottom left panel), and percentage of mitochondrial RNA (bottom right panel) in distinct batches of single-cell data from gout patients. (**D**) tSNE of the total cells profiled here, with each cell color-coded for: the clusters (left), the group (median), the corresponding patient(right).

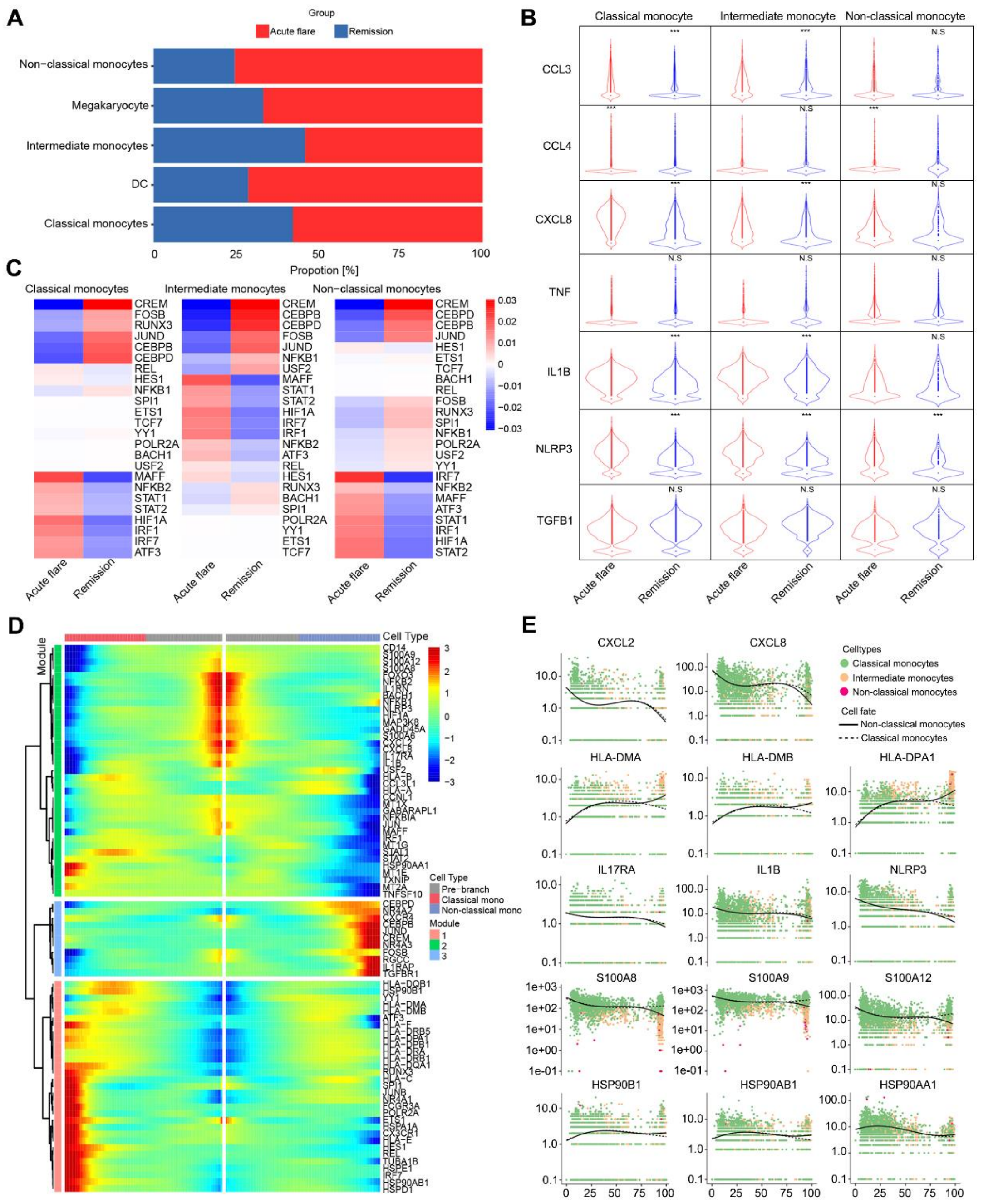

Supplemental Figure 2 Detailed characterization of monocyte subtypes from the gout flare and remission of gout. (**A**) Bar plot of cell fractions of myeloid subtypes stratified by groups. (**B**) Violin plots of the average expression of genes involved in the inflammatory cytokines in each monocyte subtypes from gout flare and remission. *P* values were calculated using two-sided Wilcoxon rank-sum tests. Data are from single-cell transcriptomes of 3 independent gout patients. *** $P < 0.001$ (**C**) Heatmap of the t values of AUC scores of expression regulation by transcription factors in each monocyte subtypes from gout flare and remission, as estimated using SCENIC. (**D**) Gene expression changes of selected marker genes as a function of pseudotime reflecting the cellular differentiation. (**E**) DEG analysis as a function of pseudotime in a branch-dependent manner showing a common gene signature of a pre-branch precursor cell population choosing two main cell fates.

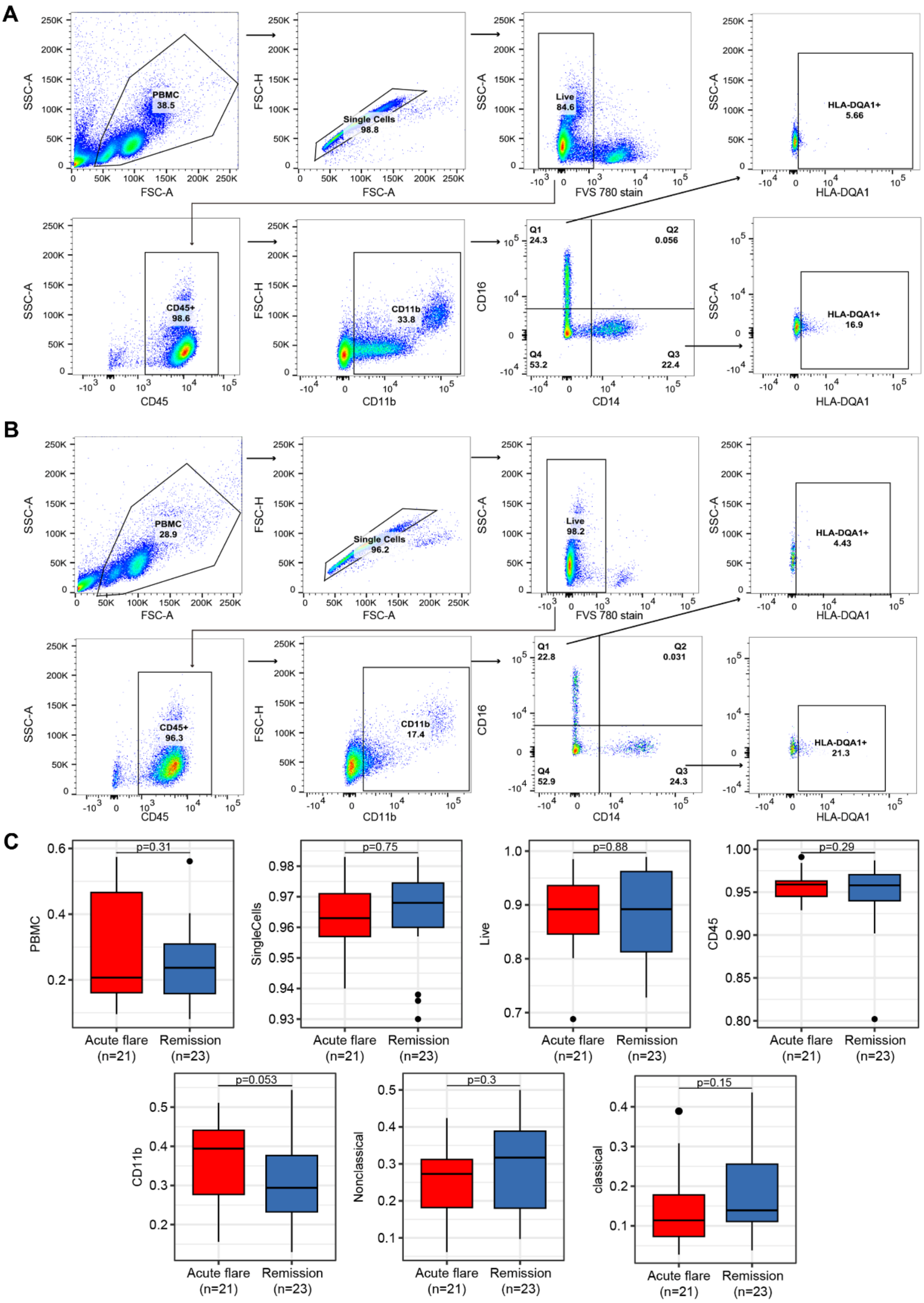

Supplemental Figure 3 Flow cytometry of monocyte subtypes from the gout flare and remission of gout. (**A-B**) Percentages of PBMCs, single cells, live cells, CD45$^+$ cells, CD11b$^+$ cells, classical monocytes, HLA-DQA1$^+$ classical monocytes, non-classical monocytes and HLA-DQA1$^+$ non-classical monocytes in gout flare (A) and gout remission patients (B). Cells were gated on lymphocytes and one representative flow cytometry chart from each group is shown. (**C**) Box plots of the percentages of monocyte subtypes. The box represents the interquartile range (IQR). The horizontal line inside the box represents the median. The whiskers extend from the box, indicating

the data range without outliers. Outliers are shown as individual points beyond the whiskers and defined as values outside 1.5 times the IQR range. Statistical analysis was undertaken using a 2-tailed Student's t test.

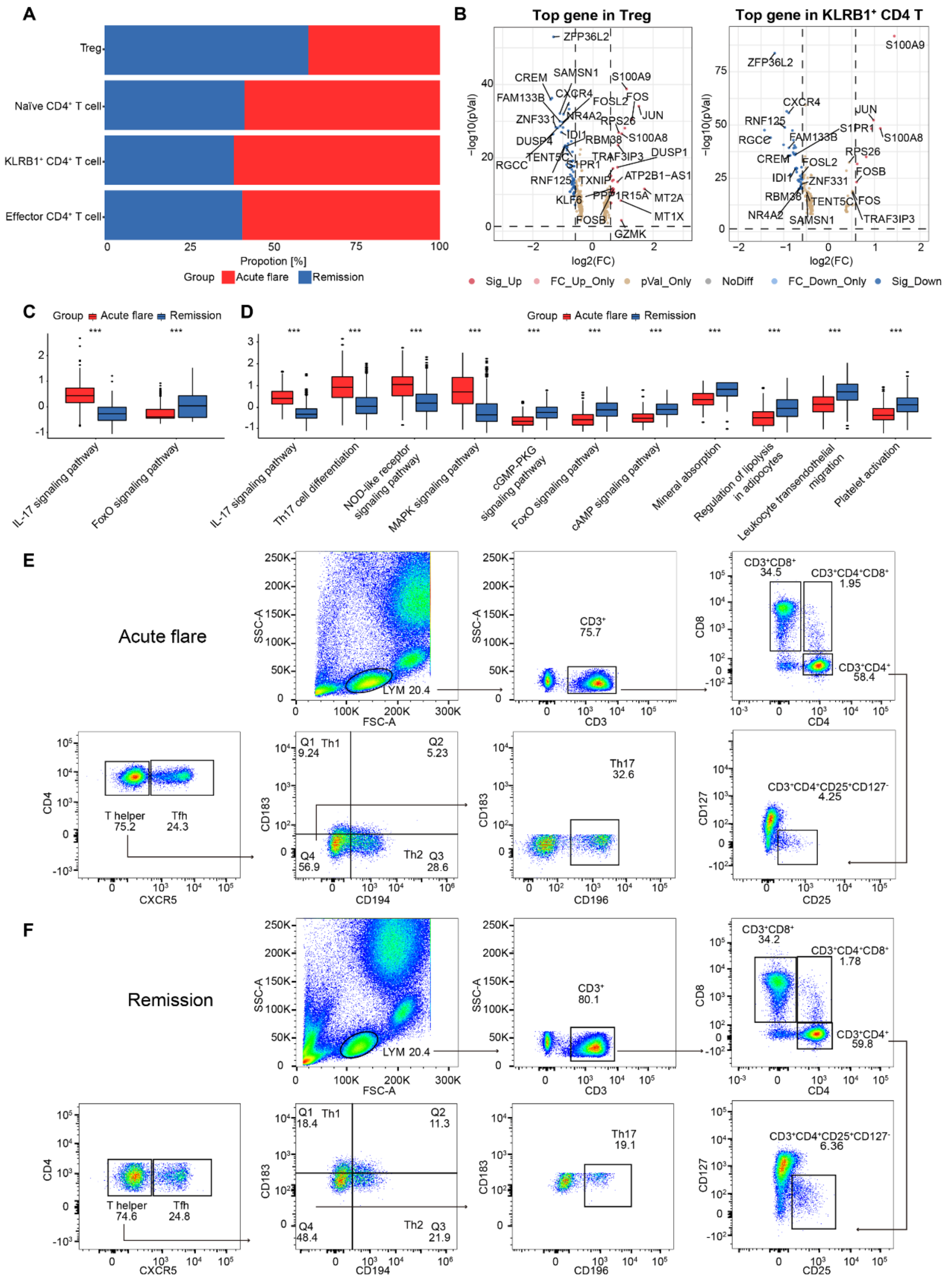

Supplemental Figure 4 The role of CD4$^+$ T cell subtypes in gout flare and remission. (**A**) Bar plot of cell fractions of CD4$^+$ TC subtypes stratified by groups. (**B**) The volcano plot represents the top differentially expressed genes of KLRB1$^+$ CD4$^+$ TC and Treg cells between gout flare and remission. (**C-D**) Box plots of the expression of genes involved in the top KEGG pathways term of KLRB1$^+$ CD4$^+$ TCs (**C**) and Treg cells (**D**) between gout flare and remission. *P* values were

determined by the Wilcoxon rank-sum tests. Data are from single-cell transcriptomes of 3 independent gout patients. *** $P < 0.001$ (**E-F**) Percentages of TCs, CD4^{+} TCs, Th1 cells, Th2 cells, Th17 cells, and Treg in gout flare patients (**E**) and gout remission patients (**F**). Cells were gated on lymphocytes and one representative flow cytometry chart from each group is shown.

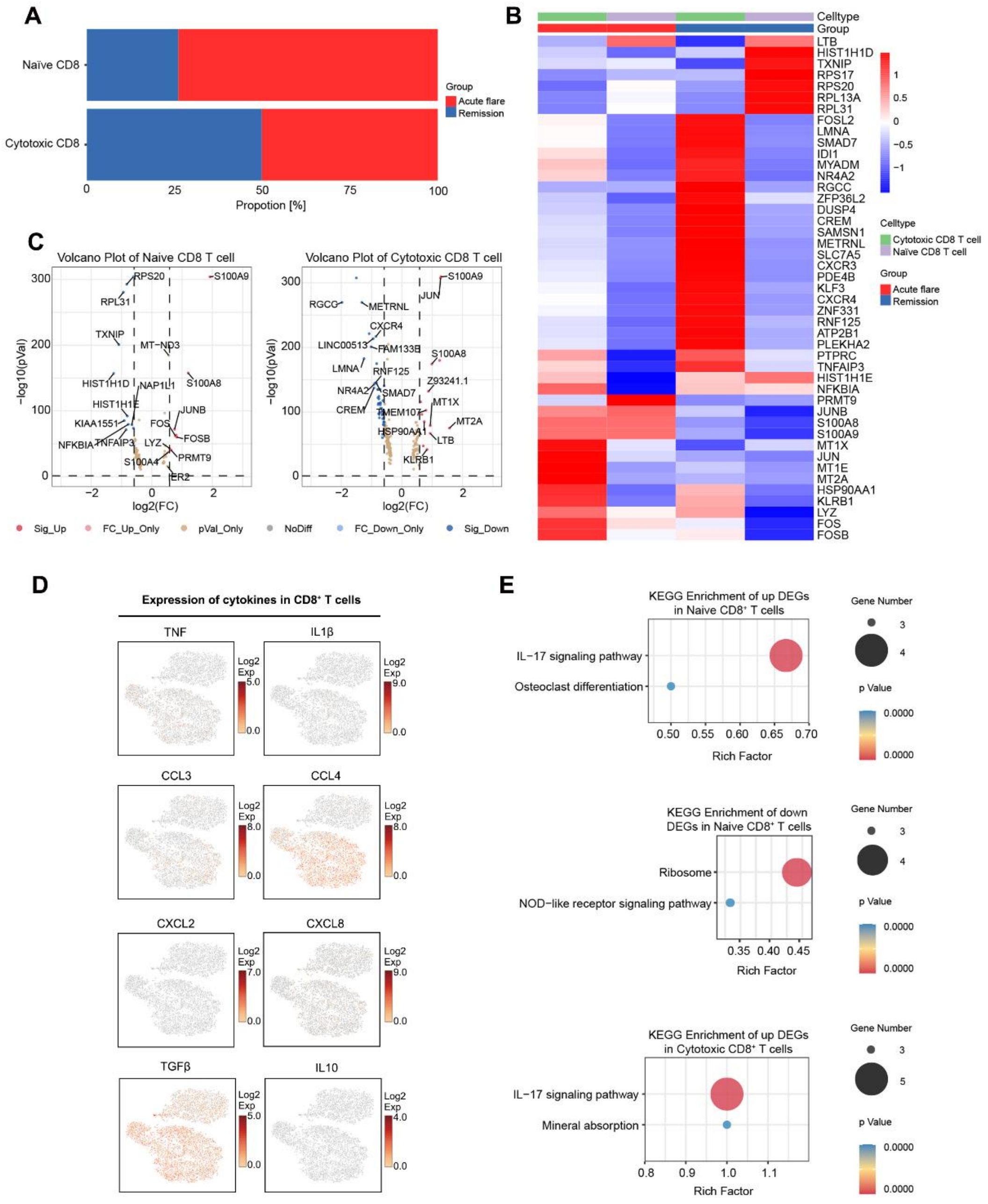

Supplemental Figure 5 The role of CD8^{+} T cell subtypes in gout flare and remission. (**A**) Bar plot of cell fractions of CD8^{+} TC subtypes stratified by groups. (**B**) Heatmaps of DE genes between CD8^{+} TC subtypes of gout flare compared to remission of gout. The heatmap is colored by average log(FC). All displayed genes and regulators are statistically significant at the $P < 0.05$. (**C**) The volcano plot represents the top differentially expressed genes of naïve CD8^{+} TCs and cytotoxic CD8^{+} TCs between gout flare and remission. (**D**) tSNE plots showing the expression of selected cytokines in CD8^{+} TC subtypes. (**E**) Naïve CD8^{+} TCs and cytotoxic CD8^{+} TCs functional enrichment analysis with KEGG of each monocyte subtypes were performed with up and down

regulated genes.

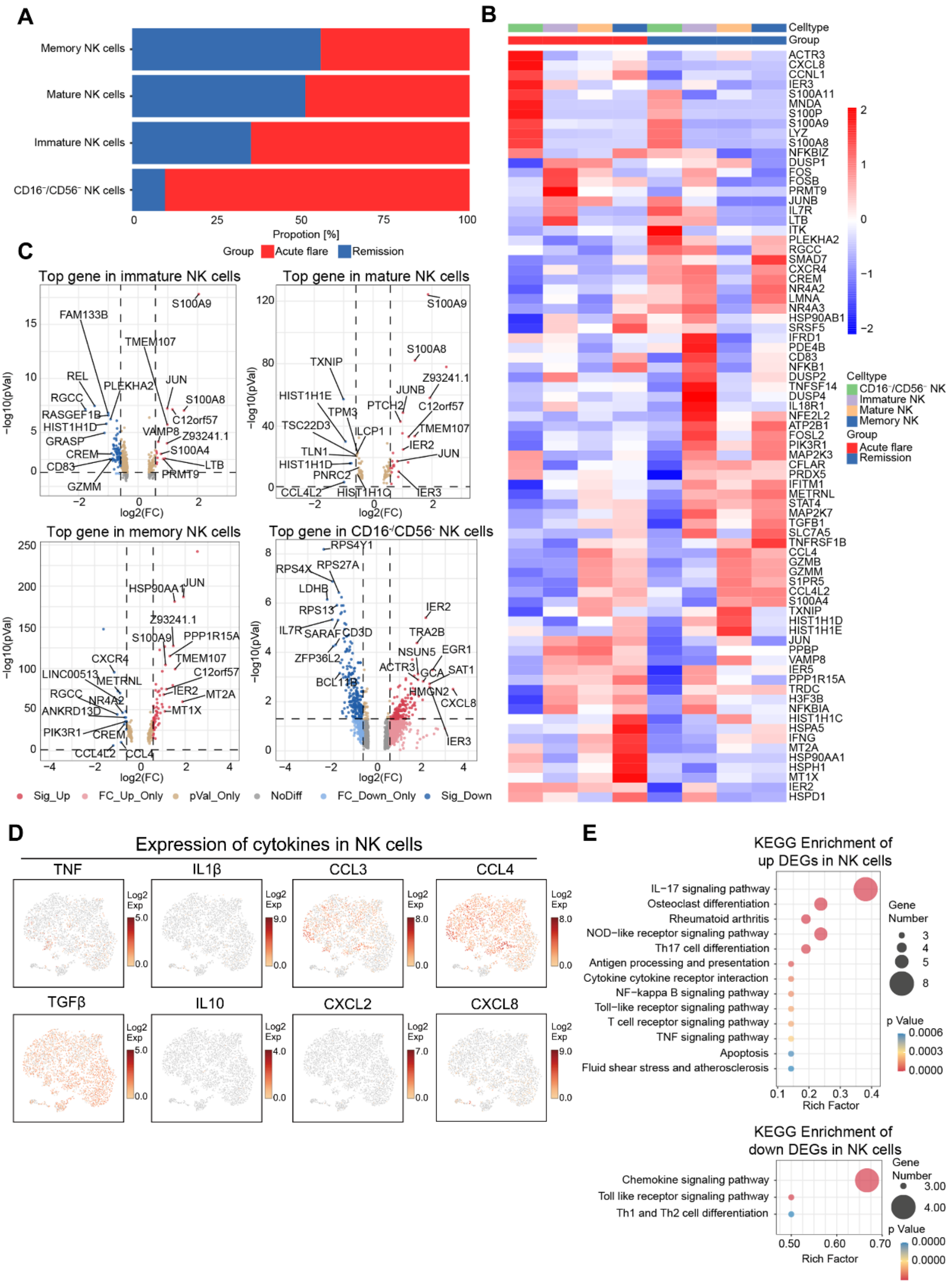

Supplemental Figure 6 The role of NK cells subtypes in gout flare and remission. (**A**) Bar plot of cell fractions of NK cell subtypes stratified by groups. (**B**) Heatmaps of DE genes between NK cell subtypes of gout flare compared to remission of gout. The heatmap is colored by average log(FC). All displayed genes and regulators are statistically significant at the $P < 0.05$. (**C**) The volcano plot represents the top differentially expressed genes of NK cell subtypes between gout flare and remission. (**D**) tSNE plots showing the expression of selected cytokines in NK cell subtypes. (**E**) NK cells functional enrichment analysis with KEGG of each monocyte subtypes were

performed with up and down regulated genes.

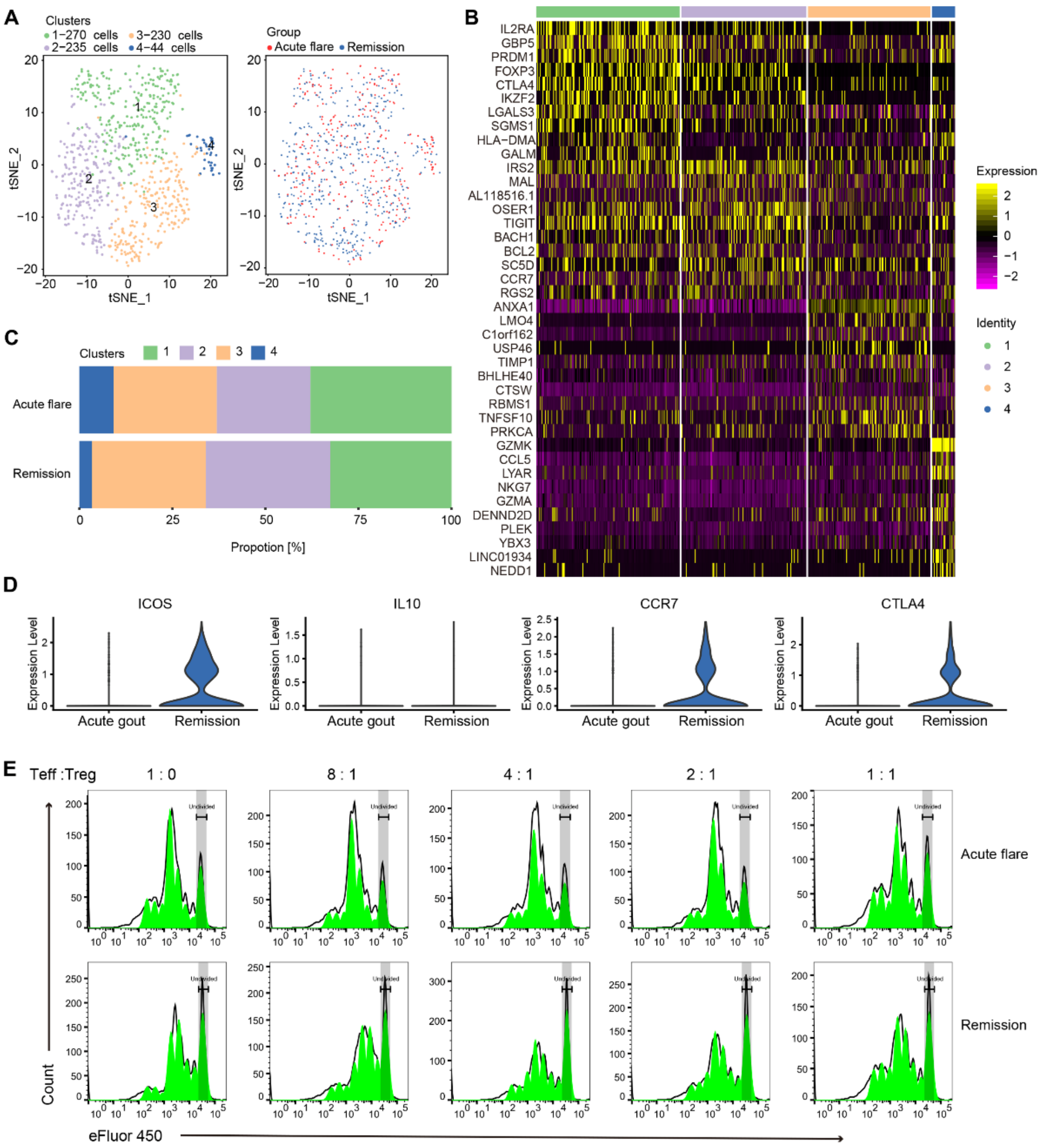

Supplemental Figure 7 The role of Treg cell subtypes in gout flare and remission. (**A**) t-SNE representations of integrated single-cell transcriptomes of Treg cells, cells are color-coded by clusters and disease state. (**B**) Heatmap of all clusters from Treg cells after dimensional reduction. Top 10 marker genes were used and colored by their expression level. (**C**) Bar plot showing cell fractions of Treg subtypes in patients with gout flare and gout remission, color coded for cell clusters. (**D**) Violin plots of the average expression of genes involved in the function in Treg cells from gout flare and remission. (**E**) Treg cell suppression assay. Sorted Tregs ($CD4^+CD25^+CD127^-$) were co-cultured with eFlour 450-labeled Teffs ($CD4^+CD25^-$) at different Treg-to-Teff ratios in the presence of anti-CD3/CD28 beads. The percentages of divided T cells are shown in each histogram plot.

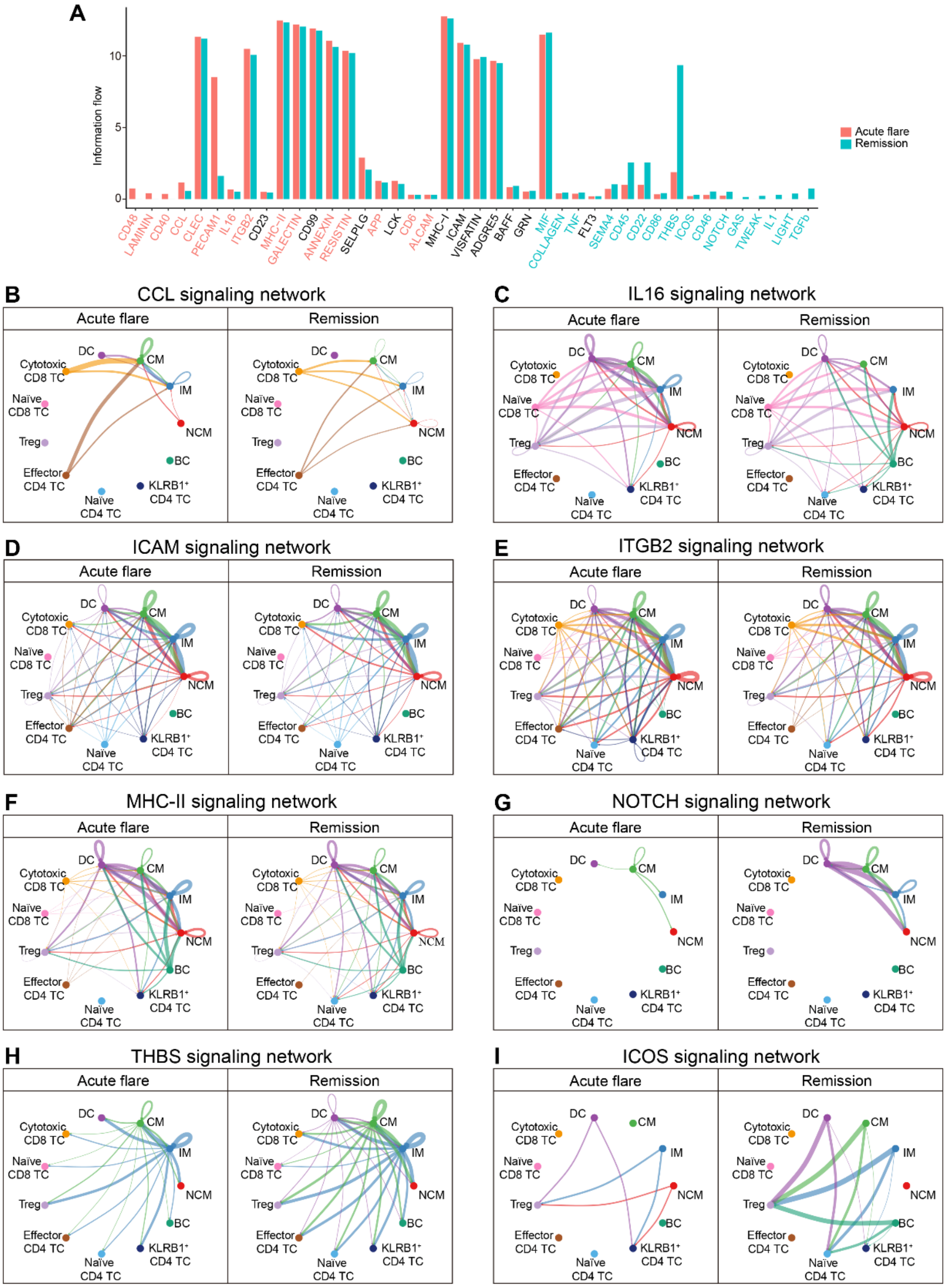

Supplemental Figure 8 Some cell–cell communications mediated by signaling pathways are greatly altered in gout flare compared with remission. (**A**) Significant signaling pathways were ranked based on differences in the overall information flow within the inferred networks between gout flare and remission. The overall information flow of a signaling network is calculated by summarizing all communication probabilities in that network. Red-colored labels are more enriched in the gout flare, black-colored labels are equally enriched in gout flare and remission, blue-colored labels are enriched in gout remission. Circle plots show and compare cell–cell communication alterations between gout flare and remission mediated by some of the signaling axes, including C-

C Motif Chemokine Ligand (CCL) (**B**), IL16 (**C**), Intercellular Adhesion Molecule (ICAM) (**D**), Integrin Subunit (ITGB2) (**E**), Class II Major Histocompatibility Complex (MHC-II) (**F**), NOTCH (**G**), Thrombospondin (THBS) (**H**) and Inducible T Cell Costimulator (ICOS) (**I**).

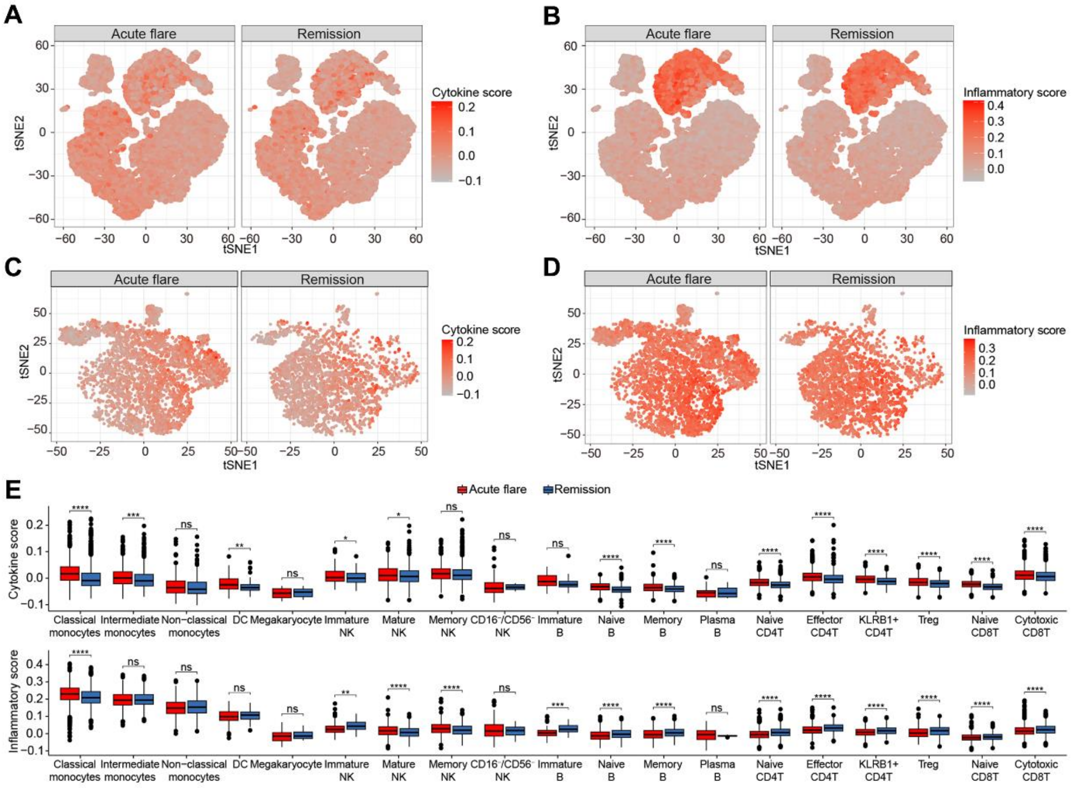

Supplemental Figure 9 Infammatory score and cytokine score of cell subtypes between gout flare and remission. (**A-B**) t-SNE plots of PBMCs colored by cytokine score (A), and inflammatory score (B). (**C-D**) t-SNE plots of myeloid cells colored by cytokine score (C), and inflammatory score (D). (**E**) Boxplots of the cytokine score (top panel) and inflammatory score (bottom panel) of cell subtypes. Significance was evaluated with the t-test. Data are from single-cell transcriptomes of 3 independent gout patients. * $P < 0.05$,** $P < 0.001$,*** $P < 0.001$,****$P < 0.0001$.

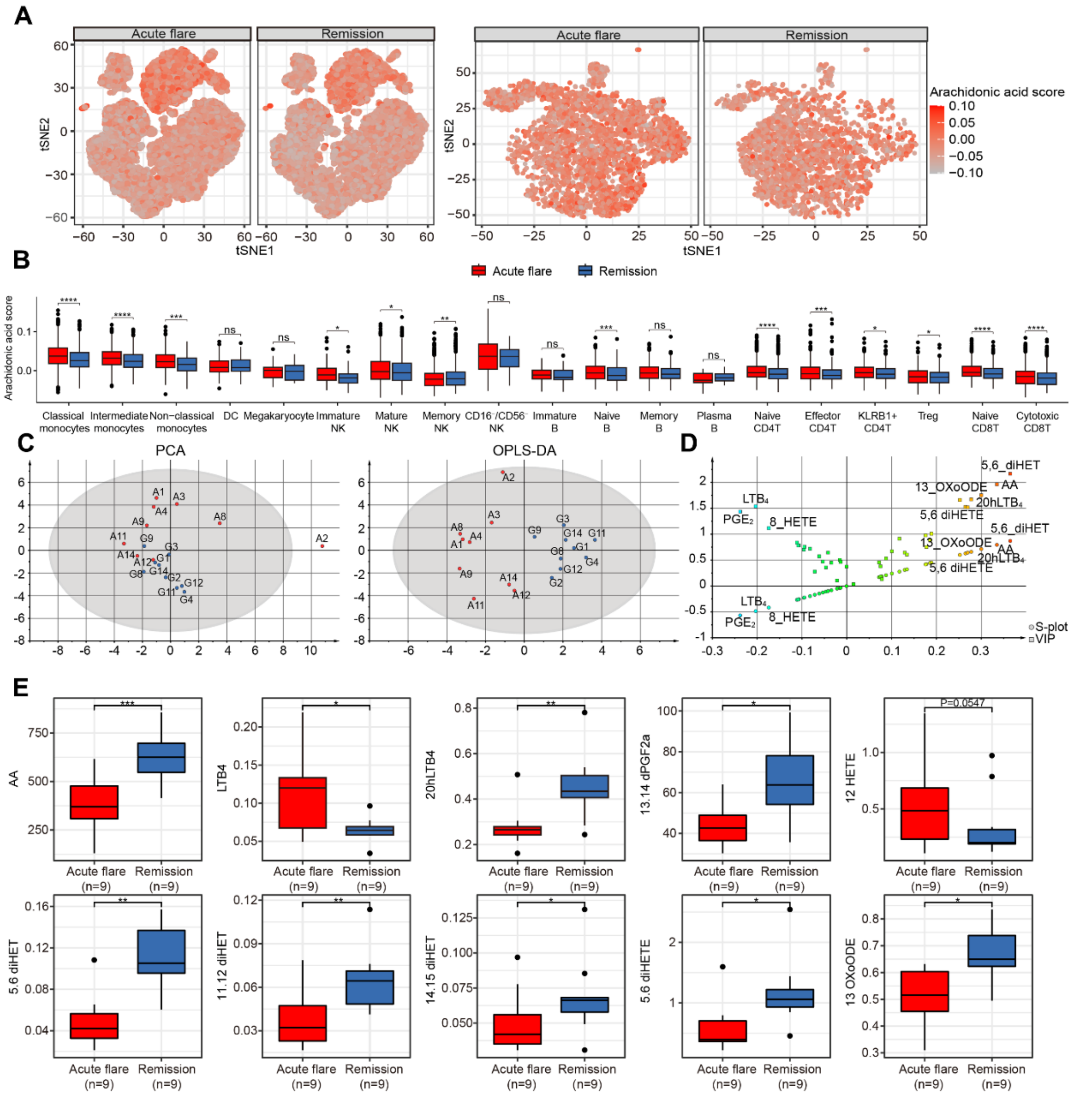

Supplemental Figure 10 Detailed characterization of arachidonic acid metabolic activity for the gout flare and remission. (**A**) t-SNE plots of PBMCs (left panel) and myeloid cells (right panel) colored by arachidonic acid score in gout flare and remission. (**B**) Boxplots of the arachidonic acid score of cell subtypes. Significance was evaluated with the t-test. Data are from single-cell transcriptomes of 3 independent gout patients. * $P < 0.05$,** $P < 0.001$,*** $P < 0.001$,****$P < 0.0001$. (**C**) 2D PCA score plots and 2D scatter plot for the orthogonal projections to latent structures discriminant analysis (OPLS-DA) show visual separation of gout flare (red dots) from the remission (blue dots). (**D**) S-plot and VIP value of the analysis of OPLS-DA. (**E**) Plasma levels of ten significant expressed metabolites from gout flare (n = 9) and remission (n = 9) patients. The box represents the interquartile range (IQR). The horizontal line inside the box represents the median. The whiskers extend from the box, indicating the data range without outliers. Outliers are shown as individual points beyond the whiskers and defined as values outside 1.5 times the IQR range. Statistical analysis was undertaken using a 2-tailed Student's t test. * $P < 0.05$,** $P < 0.001$,*** $P < 0.001$.

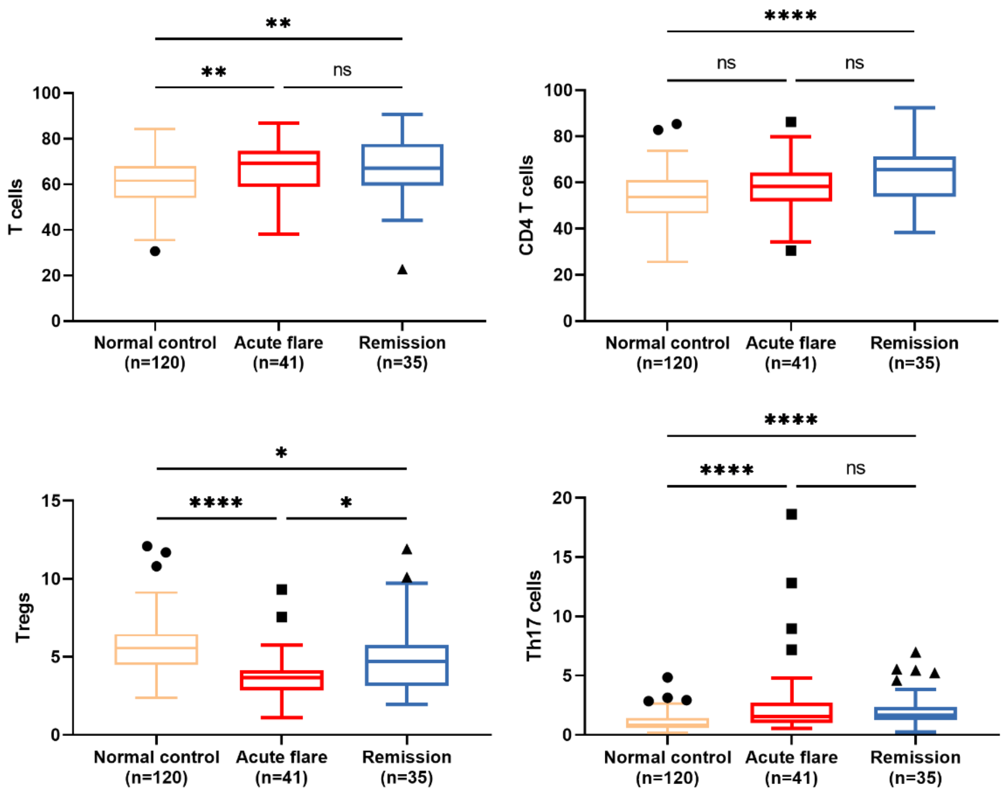

Supplemental Figure 11 Flow cytometry of CD4^{+} T cells subtypes in normal control, gout flare and remission. Boxplots of the percentages of T cells, CD4^{+} T cells, Th17 cells and Treg in normal control (n = 120), gout flare (n = 41) and remission (n = 35) patients. The box represents the interquartile range (IQR). The horizontal line inside the box represents the median. The whiskers extend from the box, indicating the data range without outliers. Outliers are shown as individual points beyond the whiskers and defined as values outside 1.5 times the IQR range. Statistical analysis was done using the Kruskal-Wallis test followed by Dunn's multiple comparison correction. * $P < 0.05$,** $P < 0.001$,**** $P < 0.0001$

Supplemental Table 1 Percentages of monocyte subtypes between gout flare and remission by flow cytometry

Number	Sample ID	group	PBMC	SingleCells	Live	CD45+	CD11b+	Nonclassical	Nonclassical+,HLA-DQA1+	classical	Classical+,HLA-DQA1+
Specimen_001_A11_066.fcs	AG11	Acute flare	39.00%	95.50%	97.10%	98.20%	39.40%	26.30%	7.14%	38.90%	8.41%
Specimen_001_A14_017.fcs	AG14	Acute flare	31.50%	97.50%	98.50%	96.30%	15.60%	17.30%	5.98%	30.80%	19.10%
Specimen_001_A16_057.fcs	AG16	Acute flare	55.90%	97.10%	96.70%	98.30%	32.40%	6.18%	8.04%	7.87%	9.01%
Specimen_001_A21_070.fcs	AG21	Acute flare	57.40%	98.20%	86.80%	95.60%	27.10%	27.50%	3.10%	2.77%	5.34%
Specimen_001_A23_071.fcs	AG23	Acute flare	16.10%	96.00%	89.10%	96.10%	27.20%	42.40%	3.01%	19.00%	5.02%
Specimen_001_A24_072.fcs	AG24	Acute flare	20.70%	96.40%	89.70%	92.90%	28.40%	18.20%	6.12%	7.34%	10.50%
Specimen_001_A33_078.fcs	AG33	Acute flare	16.40%	96.20%	92.30%	94.00%	27.70%	38.20%	10.30%	11.40%	10.50%
Specimen_001_A36_080.fcs	AG36	Acute flare	18.20%	94.00%	87.40%	96.10%	51.10%	30.40%	15.00%	13.00%	10.20%
Specimen_001_A40_019.fcs	AG40	Acute flare	55.80%	97.90%	97.40%	99.10%	45.60%	27.30%	2.05%	22.40%	11.40%
Specimen_001_A41_083.fcs	AG41	Acute flare	34.70%	95.70%	68.80%	96.70%	44.10%	23.70%	9.84%	3.08%	16.10%
Specimen_001_A42_020.fcs	AG42	Acute flare	47.20%	98.30%	90.40%	98.40%	41.20%	19.20%	3.13%	9.66%	21.80%
Specimen_001_A43_021.fcs	AG43	Acute flare	12.40%	96.10%	89.20%	96.00%	40.00%	29.50%	3.25%	8.28%	13.10%
Specimen_001_A44_084.fcs	AG44	Acute flare	24.60%	94.40%	80.10%	95.90%	47.20%	22.60%	12.90%	5.81%	15.80%
Specimen_001_A49_087.fcs	AG49	Acute flare	10.50%	97.70%	93.60%	94.00%	25.40%	16.80%	6.69%	20.40%	17.40%
Specimen_001_A50_088.fcs	AG50	Acute flare	11.80%	96.80%	93.10%	94.50%	35.70%	31.20%	4.24%	17.80%	15.70%
Specimen_001_A53_090.fcs	AG53	Acute flare	9.60%	94.80%	94.50%	93.00%	45.80%	36.30%	3.29%	16.80%	17.40%
Specimen_001_A54_091.fcs	AG54	Acute flare	19.00%	96.90%	84.60%	94.80%	46.80%	28.80%	7.65%	10.70%	9.18%
Specimen_001_A55_092.fcs	AG55	Acute flare	13.70%	96.30%	80.70%	94.00%	39.60%	34.90%	5.40%	6.32%	14.40%
Specimen_001_A56_093.fcs	AG56	Acute flare	16.60%	96.80%	84.60%	96.30%	29.80%	39.50%	6.24%	17.40%	5.73%
Specimen_001_A58_095.fcs	AG58	Acute flare	46.60%	96.20%	80.90%	95.00%	25.80%	18.20%	9.93%	6.27%	14.80%
Specimen_001_A59_096.fcs	AG59	Acute flare	50.60%	95.70%	82.90%	95.30%	41.10%	11.10%	9.23%	17.70%	11.10%
Specimen_001_G1_027.fcs	RG1	Remission	15.50%	97.90%	94.70%	94.20%	13.00%	17.30%	3.85%	27.20%	24.70%
Specimen_001_G2_028.fcs	RG2	Remission	26.00%	97.10%	72.80%	94.90%	14.60%	14.40%	3.68%	14.70%	20.30%
Specimen_001_G3_029.fcs	RG3	Remission	15.70%	98.20%	97.30%	96.20%	25.20%	30.30%	5.83%	24.10%	8.45%
Specimen_001_G4_026.fcs	RG4	Remission	28.90%	96.20%	98.20%	96.30%	17.40%	22.80%	4.43%	24.30%	21.30%
Specimen_001_G5_031.fcs	RG5	Remission	22.30%	97.50%	89.20%	93.80%	20.70%	37.90%	10.20%	12.90%	28.60%
Specimen_001_G6_030.fcs	RG6	Remission	30.10%	97.30%	93.40%	98.70%	38.60%	38.60%	2.90%	10.80%	24.70%
Specimen_001_G7_032.fcs	RG7	Remission	31.70%	98.10%	85.80%	98.60%	24.90%	45.30%	5.01%	13.90%	8.33%
Specimen_001_G9_033.fcs	RG9	Remission	31.90%	97.10%	77.40%	97.50%	32.90%	32.80%	5.87%	8.91%	15.50%
Specimen_001_G10_045.fcs	RG10	Remission	23.70%	97.10%	89.90%	91.00%	41.10%	43.90%	2.31%	8.70%	7.50%
Specimen_001_G11_050.fcs	RG11	Remission	11.40%	96.30%	96.20%	96.20%	23.30%	9.69%	4.48%	18.20%	8.73%
Specimen_001_G12_034.fcs	RG12	Remission	23.30%	95.90%	80.80%	95.80%	25.10%	10.30%	8.04%	26.00%	13.50%
Specimen_001_G13_035.fcs	RG13	Remission	26.10%	96.20%	78.10%	96.20%	40.30%	37.70%	5.22%	3.82%	13.90%
Specimen_001_G14_058.fcs	RG14	Remission	8.08%	93.60%	81.80%	90.20%	36.70%	40.10%	6.45%	12.80%	18.90%
Specimen_001_G15_059.fcs	RG15	Remission	11.10%	93.80%	88.80%	97.90%	25.20%	31.70%	4.66%	11.40%	8.34%
Specimen_001_G19_051.fcs	RG19	Remission	8.50%	95.80%	96.10%	95.60%	40.10%	39.80%	3.89%	33.60%	11.50%
Specimen_001_G20_060.fcs	RG20	Remission	23.60%	97.40%	79.40%	92.70%	31.90%	18.80%	2.75%	28.00%	8.11%
Specimen_001_G21_061.fcs	RG21	Remission	40.30%	93.00%	80.30%	94.40%	36.00%	10.10%	7.75%	9.33%	7.91%
Specimen_001_G22_062.fcs	RG22	Remission	21.30%	95.70%	85.20%	92.40%	54.40%	25.60%	4.55%	13.10%	12.80%
Specimen_001_G23_063.fcs	RG23	Remission	16.00%	96.10%	98.90%	80.20%	23.20%	50.00%	5.64%	29.10%	9.78%
Specimen_001_R14_022.fcs	R14	Remission	23.70%	96.80%	96.20%	96.60%	18.10%	36.60%	3.81%	25.10%	12.50%
Specimen_001_R16_054.fcs	RG16	Remission	56.10%	96.70%	98.50%	97.90%	29.40%	27.70%	6.39%	13.10%	6.74%
Specimen_001_R42_024.fcs	RG42	Remission	33.90%	98.30%	86.60%	98.00%	47.40%	39.10%	3.06%	6.15%	11.70%
Specimen_001_R43_025.fcs	RG43	Remission	32.50%	98.20%	98.00%	94.40%	31.00%	16.20%	0.17%	43.60%	2.51%

Supplemental Table 2 Percentages of Treg cells between gout flare and remission by flow cytometry

Sample ID	Group	T cells (% of lymphocyte)	$CD4^+$ T cells (% of T cells)	Th1 (% of Th cells)	Th2 (% of Th cells)	Th17 (% of Th cells)	Treg cells (% of T cells)
AG1	Acute flare	54.6	51.7	8.64	27.7	1.01	3.23
A2	Acute flare	65.9	51	28	9.08	1.26	3.39
AG4	Acute flare	73.3	79.9	38.3	13.4	1.3	2.2
AG7	Acute flare	76.6	30.5	16.5	11.7	1.51	1.1
A8	Acute flare	65.3	54.6	26.2	10.9	2.24	2.11
A9	Acute flare	54.5	65.9	13.6	21.2	2.53	2.84
A10	Acute flare	72.2	38.7	30.9	13.6	0.86	1.62
A13	Acute flare	61.7	65.8	27.5	9.35	2.6	2.62
AG12	Acute flare	75.5	43.6	41.3	8.74	0.6	2.52
AG11	Acute flare	58.3	62.7	31.6	21.4	2.28	3.71
AG14	Acute flare	61.8	62.4	26.3	20	2.42	5.66
AG16	Acute flare	83.4	55.7	30.5	5.7	0.62	2.82
AG21	Acute flare	71.6	64.6	20.7	16.6	0.9	4.79
AG23	Acute flare	74.8	47.4	24.5	13.2	1.17	3.98
AG24	Acute flare	45.2	58.5	13.8	6.47	0.54	9.32
AG33	Acute flare	59.4	57.5	15.1	11.9	0.76	5.74
AG36	Acute flare	48.8	54.7	23.7	15.4	1.75	4.77
AG40	Acute flare	65.8	52.8	11.4	19.9	0.62	3.7
AG41	Acute flare	72.2	59.4	55.5	9.28	1.14	3.76
AG42	Acute flare	64.2	58.4	7.65	24.2	1.28	5.63
AG43	Acute flare	74.7	48.3	17.6	18.5	1.39	2.9
AG44	Acute flare	75.5	51.6	20.2	16.4	1.23	3.04
AG49	Acute flare	74.9	59.6	19.1	25.7	1	3.73
AG50	Acute flare	68	52.1	8.89	42.4	0.52	4.28
A52	Acute flare	78.7	79.4	15.9	17.7	1.39	7.55
AG53	Acute flare	47.7	55.9	21.4	23.4	7.18	3
AG59	Acute flare	57.8	57.5	9.57	18.5	12.8	4.1
A1	Acute flare	75.7	58.4	9.24	28.6	18.6	2.48
AG3	Acute flare	69.4	64.2	29.5	11	4.51	3.68
A3	Acute flare	64.3	57.4	17.5	15	4.67	4.05
AG5	Acute flare	58.1	64.4	17.6	10.6	1.9	4.16
AG6	Acute flare	86.9	71	18.2	12.5	3.04	3.02
AG8	Acute flare	61.5	86.3	12.6	26	1.91	3.63
AG9	Acute flare	72.8	66.6	10.1	16.1	8.95	4.54
AG10	Acute flare	38.2	34.3	6.56	17.2	2.81	1.57
AG13	Acute flare	72.5	60	29	8.32	1.74	3.98
AG15	Acute flare	72.5	61.8	12.6	21.5	2.59	3.62
AG19	Acute flare	70.5	47.7	3.87	19.8	4.76	3.86
AG17	Acute flare	82.7	64.4	8.79	13	0.55	3.66
AG20	Acute flare	50.4	76.7	16.7	15.5	2.93	3.1
AG18	Acute flare	69.5	53	21.3	16.8	1.2	2.08
P2	Remission	79.7	57.5	22.3	9.08	1.53	3.12
G7	Remission	71.9	65.5	14.5	22	1.54	2.91
G8	Remission	68.7	51.9	22.7	11.3	1.15	2.62
G9	Remission	60.7	67.8	13.6	20.8	2.16	3.57
G10	Remission	66.4	72.6	22.3	8.6	2.78	4.8
G13	Remission	77.2	38.7	20.8	19.9	2.26	1.93
RG42	Remission	70.9	57.5	11.3	10.4	1.24	5.08
RG43	Remission	77.7	51.2	10	26.2	1.43	3.81
G52	Remission	75.7	80.1	10.7	12.3	1.53	7.37
G22	Remission	67.1	53.9	27.4	9.44	1.17	3.78
G23	Remission	44.1	57.2	8.69	13.9	3.11	6.41
R28	Remission	90.7	80.2	18	8.16	1.21	5.07
R29	Remission	59.4	66	39.7	17.7	4.57	2.88
R30	Remission	45.6	53.2	4.24	8.77	1.57	2.79
R31	Remission	22.7	85.3	14.4	18.7	1.66	9.72
RG9	Remission	58.8	68.9	17.4	25.3	2.22	5
RG11	Remission	83.8	38.3	27.6	18.8	1.23	2.5
RG14	Remission	63.8	68.8	10.8	17.5	1.31	6.64
RG39	Remission	55.8	79.2	26.2	17.6	0.99	6.17
RG1	Remission	78.1	66.3	35.2	19	1.75	10.1
RG2	Remission	73.6	70.9	30.3	19.6	1.32	11.9
RG6	Remission	57.9	57.1	15.2	24.4	1.03	5.41
RG13	Remission	66.8	40.6	14.8	29.6	0.81	2.67
RG56	Remission	60.8	75.8	16.2	18.2	1.79	6
RG32	Remission	54.4	71.4	11.2	13	6.95	4.21
RG33	Remission	86.1	53.6	22.4	14.2	3.83	5.67
RG34	Remission	73	65.8	19.8	22.3	2.38	4.94
RG35	Remission	78.9	70.9	16.9	12.1	2.37	3.65
RG36	Remission	77.6	65.6	8.33	13.6	1.76	5.65
RG37	Remission	63.9	49.4	9.18	22.3	5.42	3.44
RG38	Remission	82.3	76.4	6.64	12.6	0.25	2.69
RG49	Remission	65.5	59	16	15.9	5.53	4.42
RG40	Remission	44.8	53.8	11.3	23.8	5.21	4.7
RG41	Remission	82.1	92.4	4.98	11.9	2.05	4.4
RG44	Remission	64.5	63.7	18.2	9.04	0.98	5.77

Supplemental Table 3 A gene list associated with cytokine, inflammatory response and arachidonic acid pathway

Cytokines	Inflammatory	Arachidonic acid pathway
IL2	ABCA1	AKR1C3
IL7	ABI1	ALOX12
CSF3	ACVR1B	ALOX12B
CXCL10	ACVR2A	ALOX15
CCL2	ADGRE1	ALOX15B
CCL3	ADM	ALOX5
TNF	ADORA2B	CBR1
TFTN1	ADRM1	CBR3
IL6	AHR	CYP2B6
CCL7	APLNR	CYP2C18
IL1RN	AQP9	CYP2C19
CSF1	ATP2A2	CYP2C8
IFNG	ATP2B1	CYP2C9
IL2RA	ATP2C1	CYP2E1
IL10	AXL	CYP2J2
IL18	BDKRB1	CYP2U1
HGF	BEST1	CYP4A11
CXCL9	BST2	CYP4A22
CCL27	BTG2	CYP4F2
TGFB1	C3AR1	CYP4F3
IL1B	C5AR1	EPHX2
LTA	CALCRL	GGT1
CSF2	CCL17	GGT5
LTB	CCL2	GGT6
TNFSF13	CCL20	GGT7
IL4	CCL22	GPX1
CCL12	CCL24	GPX2
CXCL8	CCL5	GPX3
CXCL11	CCL7	GPX4
CCL4	CCR7	GPX5
CXCL1	CCRL2	GPX6
CXCL2	CD14	GPX7
CXCL3	CD40	HPGDS
CCL3L1	CD48	JMJD7-PLA2G4B
CCL8	CD55	LTA4H
CXCL16	CD69	LTC4S
IFNA1	CD70	PLA2G10
CCL5	CD82	PLA2G12A
CCL11	CDKN1A	PLA2G12B
IFNA2	CHST2	PLA2G1B
CCL20	CLEC5A	PLA2G2A
CCL4L2	CMKLR1	PLA2G2C
OSM	CSF1	PLA2G2D
TNFSF14	CSF3	PLA2G2E
SA100A12	CSF3R	PLA2G2F
FGF19	CX3CL1	PLA2G3
CXCL5	CXCL10	PLA2G4A
CCL19	CXCL11	PLA2G4B
IL18R1	CXCL6	PLA2G4E
TGFA	CXCL8	PLA2G5
IFNB1	CXCL9	PLA2G6
IL8	CXCR6	PTGDS
IL17C	CYBB	PTGES
TNFSF10	DCBLD2	PTGES2
FGF7	EBI3	PTGIS
XCL1	EDN1	PTGS1
FGF13	EIF2AK2	PTGS2
LIF	EMP3	TBXAS1
TGFB3	EREG	
INHBE	F3	
CERS1	FFAR2	
TXLNA	FPR1	
IFNW1	FZD5	
IL22	GABBR1	
XCL2	GCH1	
CCL25	GNA15	
CCL16	GNAI3	
CD40LG	GP1BA	
IL20	GPC3	
FASLG	GPR132	
TPO	GPR183	
SCYL3	HAS2	
PF4V1	HBEGF	
TNFSF8	HIF1A	
GDF15	HPN	
IL1A	HRH1	
VEGFA	ICAM1	
GDF7	ICAM4	
BMP6	ICOSLG	
PDGFA	IFITM1	
IL21	IFNAR1	
ABCD-1	IFNGR2	
ABCD-2	IL10	
PDGFB	IL10RA	
TNFSF4	IL12B	
FAM19A1	IL15	
HBEGF	IL15RA	
PDGFD	IL18	
IL12RB2	IL18R1	
GH1	IL18RAP	
VEGFB	IL1A	
MIP3B	IL1B	
IL27	IL1R1	
PF4	IL2RB	
BMP8B	IL4R	
TNFSF12	IL6	
IL15	IL7R	
SCYL2	INHBA	
SCYL1	IRAK2	
TSLP	IRF1	
GDF11	IRF7	
SDF1B	ITGA5	
INHBA	ITGB3	
PPBP	ITGB8	
FGF11	KCNA3	
IFNG-AS1	KCNJ2	
FGF22	KCNMB2	
VEGFC	KIF1B	
CCL18	KLF6	
TNFSF11	LAMP3	
IL12A	LCK	
EBI3	LCP2	
AMH	LDLR	
IL26	LIF	
IL32	LPAR1	
PDGFC	LTA	
FGF23	LY6E	
IGF1	LYN	
IL1F11	MARCO	
CCL28	MEFV	
CLCF1	MEP1A	
TNFSF9	MET	
BMP3	MMP14	
IL24	MSR1	
GDF10	MXD1	
CXCL6	MYC	
GDF9	NAMPT	
IL23A	NDP	
IL16	NFKB1	
CD70	NFKBIA	
IL5	NLRP3	
FGF9	NMI	
IFNL1	NMUR1	
TSC1	NOD2	
FGF2	NPFFR2	
IL23R	OLR1	
IL1G	OPRK1	
SPP1	OSM	
IL12RB1	OSMR	
BMP4	P2RX4	
IL13	P2RX7	
TPAR1	P2RY2	
TGFB2	PCDH7	
FAM19A2	PDE4B	
AGIF3	PDPN	
EDA	PIK3R5	
MIF	PLAUR	
TNFSF13B	PROK2	
BMP7	PSEN1	
FGF18	PTAFR	
CCL23	PTGER2	
	PTGER4	
	PTGIR	
	PTPRE	
	PVR	
	RAF1	
	RASGRP1	
	RELA	
	RGS1	
	RGS16	
	RHOG	
	RIPK2	
	RNF144B	
	ROS1	
	RTP4	
	SCARF1	
	SCN1B	
	SELE	
	SELENOS	
	SELL	
	SEMA4D	
	SERPINE1	
	SGMS2	
	SLAMF1	
	SLC11A2	
	SLC1A2	
	SLC28A2	
	SLC31A1	
	SLC31A2	
	SLC4A4	
	SLC7A1	
	SLC7A2	
	SPHK1	
	SRI	
	STAB1	
	TACR1	
	TACR3	
	TAPBP	
	TIMP1	
	TLR1	
	TLR2	
	TLR3	
	TNFAIP6	
	TNFRSF1B	
	TNFRSF9	
	TNFSF10	
	TNFSF15	
	TNFSF9	
	TPBG	
	VIP	

Supplemental Table 4 Plasma levels (ng/ml) of arachidonic acid metabolites from gout flare and remission.

Sample number	volume (uL)	9-HOTγE		13-HOTγE		13-HOTγEg		14_HDHA		16_HDHA		17_HDHA		11,12 diHETE		8,9 diHETE	
		peak area	concentration	peak area	concentration	peak area	concentration	peak area	concentration	peak area	concentration	peak area	concentration	peak area	concentration	peak area	concentration
A1	500	943	0.036669375	1800	0.114991071	5670	0.104964683	0	0	0	0	0	0	611	0.005988637	20200	0.80295
A2	500	8230	0.395099638	3360	0.265	8290	0.189465287	0	0	0	0	0	0	1500	0.018150685	54700	2.684351852
A3	500	728	0.032632753	1870	0.137709448	5390	0.115021605	0	0	0	0	0	0	472	0.005332849	18900	0.866023055
A4	500	1440	0.064270476	3830	0.28083265	6300	0.133862181	0	0	0	0	0	0	245	0.002756196	43300	1.975523673
A6	500	1600	0.063325589	4290	0.278943566	4460	0.084035432	0	0	0	0	0	0	5580	0.055665795	28000	1.132824427
A8	500	3840	0.167777235	3550	0.254817917	4360	0.09068942	0	0	0	0	0	0	2730	0.030064876	20500	0.915589888
A9	500	1110	0.048294618	784	0.056039161	8640	0.178960765	0	0	0	0	0	0	8900	0.097602452	44100	1.961370629
A11	500	761	0.029928846	1050	0.06784134	3360	0.06290902	0	0	0	0	0	0	9440	0.09357768	46100	1.853324905
A12	500	952	0.042920983	1000	0.074068323	3060	0.065678385	0	0	0	0	0	0	588	0.006681977	17200	0.792695652
A14	500	473	0.021356187	2100	0.155769231	5980	0.128538163	0	0	0	0	0	0	8690	0.09889568	57700	2.663076923
G1	500	936	0.048049074	1430	0.120599364	5420	0.132457572	0	0	0	0	0	0	2400	0.031053845	25300	1.327623762
G2	500	1860	0.091699166	4880	0.39525017	2680	0.062900712	0	0	0	0	0	0	8690	0.107985932	43000	2.16703645
G3	500	637	0.030393005	1030	0.080736744	4410	0.100170795	0	0	0	0	0	0	5180	0.062295823	23500	1.146165644
G4	500	964	0.04948644	3180	0.268185997	1880	0.045944693	0	0	0	0	0	0	12600	0.163032687	69100	3.626039604
G6	500	4070	0.245372851	2430	0.240678987	5420	0.155560637	0	0	0	0	0	0	7640	0.116096846	34700	2.138488372
G8	500	635	0.032016243	2350	0.194654434	5660	0.135856804	0	0	0	0	0	0	4380	0.055662885	27600	1.422495948
G9	500	1180	0.053982737	1160	0.087182773	10100	0.219969561	0	0	0	0	0	0	580	0.006687994	45200	2.113764706
G11	500	546	0.029798856	2800	0.251052632	5690	0.147838497	0	0	0	0	0	0	1210	0.016645133	82200	4.585894737
G12	500	1580	0.07814267	1770	0.143815012	4760	0.112074403	0	0	0	0	0	0	2710	0.033782782	16100	0.813958665
G14	500	878	0.045827911	1960	0.16807047	5720	0.142134628	0	0	0	0	0	0	840	0.011051209	56800	3.030604027

Sample number	14,15 diHETE		11_βPGF2α		15_kPGF2α		15_kPGE2		TB2		11_DTB2		12_HHTrE		14,15_diHET		11,12_diHET	
	peak area	concentration	peak area	concentration	peak area	concentration	peak area	concentration	peak area	concentration	peak area	concentration	peak area	concentration	peak area	concentration	peak area	concentration
A1	0	0	0	0	60600	0.392391855	2970	0.054348721	1920000	15.09626374	63900	1.69335	2420	0.180365625	10100	0.0578124	7470	0.050185775
A2	0	0	0	0	54200	0.433273002	810	0.018299233	2120000	20.57875458	15100	0.494012346	3260	0.299965278	11000	0.077733333	8090	0.067100156
A3	0	0	0	0	50400	0.37619101	3660	0.077204832	1270000	11.51071983	15900	0.485706052	1540	0.132309078	5810	0.038335954	4590	0.035547023
A4	0	0	0	0	95600	0.710497348	3140	0.06595075	876000	7.905503965	16600	0.504906743	1200	0.102654232	6960	0.045726301	3750	0.028916685
A6	0	0	0	0	53800	0.354565991	1050	0.019556432	1020000	8.162738025	2770	0.074712468	7470	0.566665076	4200	0.024469008	5740	0.039249973
A8	0	0	0	0	55000	0.40014744	2700	0.055514526	1140000	10.07124336	7060	0.210213483	2310	0.193446278	4760	0.030613753	2810	0.021211742
A9	0	0	0	0	62100	0.449907161	1470	0.030097759	607000	5.339995389	10800	0.320223776	2000	0.166783217	5900	0.037786406	2870	0.021573761
A11	0	0	0	0	40900	0.267845387	2340	0.043307413	1440000	11.4510218	3180	0.085228824	2400	0.18091024	5880	0.034040071	2440	0.016579192
A12	0	0	0	0	49800	0.373867401	2620	0.055587235	893000	8.140678452	8510	0.261466667	866	0.074833696	14600	0.096893217	10100	0.078672382
A14	0	0	0	0	55800	0.419519666	2100	0.044619319	1210000	11.04649197	4430	0.136307692	1200	0.103846154	5130	0.034094769	3480	0.027146262
G1	0	0	0	0	45100	0.385514986	2980	0.071989061	3470000	36.01762594	7020	0.245584158	791	0.077827351	8770	0.066269941	8260	0.073258402
G2	0	0	0	0	20800	0.170754172	3370	0.078184994	2030000	20.23601122	9360	0.314472266	900	0.085043582	9150	0.066402029	7550	0.064308386
G3	0	0	0	0	11500	0.091366627	1580	0.035475813	1110000	10.70862267	4600	0.149570552	3550	0.324645322	9640	0.067704736	5010	0.041299058
G4	0	0	0	0	57500	0.491510237	1450	0.035028234	1000000	10.37971929	19300	0.675181518	1800	0.17710396	11300	0.085387723	7330	0.06501018
G6	0	0	0	0	30900	0.310203094	597	0.016937429	958000	11.67814976	4220	0.173379845	11600	1.340406977	6780	0.060168558	5200	0.054163118
G8	0	0	0	0	38700	0.324910052	1720	0.040809958	982000	10.01116355	3100	0.106515397	748	0.072284441	4140	0.030725912	6240	0.054356244
G9	0	0	0	0	67100	0.511153048	959	0.02064584	547000	5.059838397	3250	0.101323529	800	0.070147059	7320	0.049293741	5750	0.04544739
G11	0	0	0	0	45500	0.413498452	1530	0.039295195	1350000	14.89762869	17400	0.647157895	970	0.101467105	7060	0.056717811	8070	0.076093699
G12	0	0	0	0	48700	0.40106583	1260	0.029325321	920000	9.20016073	5350	0.180317965	760	0.072042925	18000	0.131042289	13300	0.113645178
G14	0	0	0	0	47500	0.412842783	2230	0.054774885	1200000	12.66465079	4620	0.16433557	577	0.057724203	7970	0.061235275	5150	0.046442008

Sample number	8,9_diHET		12_OXoETE		5_OXoETE		12,13_DiHOME		9,10_Epome		LXB4		LXA4		Resolvin D2		Resolvin D3	
	peak area	concentration	peak area	concentration	peak area	concentration	peak area	concentration	peak area	concentration	peak area	concentration	peak area	concentration	peak area	concentration	peak area	concentration
A1	0	0	0	0	0	0	16400	0.104304	47300	1.301659615	0	0	0	0	0	0	0	0
A2	0	0	0	0	0	0	122000	0.957925926	136000	4.620512821	0	0	0	0	0	0	0	0
A3	0	0	0	0	0	0	41700	0.305719885	52700	1.671773443	0	0	0	0	0	0	0	0
A4	0	0	0	0	0	0	27300	0.199286083	46900	1.481379539	0	0	0	0	0	0	0	0
A6	0	0	0	0	0	0	19300	0.124934351	57300	1.604938344	0	0	0	0	0	0	0	0
A8	0	0	0	0	0	0	123000	0.878966292	81900	2.53238764	0	0	0	0	0	0	0	0
A9	0	0	0	0	0	0	42100	0.299587133	44000	1.354792899	0	0	0	0	0	0	0	0
A11	0	0	0	0	0	0	28500	0.183322377	46200	1.285854323	0	0	0	0	0	0	0	0
A12	0	0	0	0	0	0	31700	0.233753043	47000	1.499598662	0	0	0	0	0	0	0	0
A14	0	0	0	0	0	0	32200	0.237784615	48900	1.562485207	0	0	0	0	0	0	0	0
G1	0	0	0	0	0	0	14400	0.12090297	41200	1.496755522	0	0	0	0	0	0	0	0
G2	0	0	0	0	0	0	47700	0.384623772	44300	1.545612581	0	0	0	0	0	0	0	0
G3	0	0	0	0	0	0	31200	0.243474847	46100	1.55660925	0	0	0	0	0	0	0	0
G4	0	0	0	0	0	0	66200	0.555817822	56700	2.059855293	0	0	0	0	0	0	0	0
G6	0	0	0	0	0	0	33700	0.332297674	100000	4.266547406	0	0	0	0	0	0	0	0
G8	0	0	0	0	0	0	33400	0.275428201	38900	1.38800399	0	0	0	0	0	0	0	0
G9	0	0	0	0	0	0	17200	0.128696471	46600	1.508701357	0	0	0	0	0	0	0	0
G11	0	0	0	0	0	0	38500	0.343663158	48500	1.873238866	0	0	0	0	0	0	0	0
G12	0	0	0	0	0	0	47200	0.381802226	60400	2.114036933	0	0	0	0	0	0	0	0
G14	0	0	0	0	0	0	37100	0.316719463	43200	1.595745999	0	0	0	0	0	0	0	0

Sample number	volume (uL)	4_HDHA		7_HDHA		8_HDHA		20_HDHA		8,9_EpETE		11,12EpETE		17,18 diHETE		18cdLTB4	
		peak area	concentration	peak area	concentration	peak area	concentration	peak area	concentration	peak area	concentration	peak area	concentration	peak area	concentration	peak area	concentration
A1	500	0	0	0	0	0	0	0	0	0	0	0	0	438	0.101915122	5470	0.10592111
A2	500	0	0	0	0	0	0	0	0	0	0	0	0	2870	0.824444444	11000	0.262967975
A3	500	0	0	0	0	0	0	0	0	0	0	0	0	894	0.23979082	3640	0.081250697
A4	500	0	0	0	0	0	0	0	0	0	0	0	0	1500	0.400601883	1550	0.034449594
A6	500	0	0	0	0	0	0	0	0	0	0	0	0	290	0.068679948	4200	0.082777428
A8	500	0	0	0	0	0	0	0	0	0	0	0	0	1030	0.269284736	6140	0.13358988
A9	500	0	0	0	0	0	0	0	0	0	0	0	0	962	0.250451441	6680	0.144729014
A11	500	0	0	0	0	0	0	0	0	0	0	0	0	611	0.143786871	2030	0.039756188
A12	500	0	0	0	0	0	0	0	0	0	0	0	0	3800	1.025153765	1200	0.026941225
A14	500	0	0	0	0	0	0	0	0	0	0	0	0	560	0.151294559	576	0.012950557
G1	500	0	0	0	0	0	0	0	0	0	0	0	0	838	0.257410287	2930	0.074899717
G2	500	0	0	0	0	0	0	0	0	0	0	0	0	2790	0.823055931	2080	0.051064509
G3	500	0	0	0	0	0	0	0	0	0	0	0	0	1240	0.354020649	3500	0.083158305
G4	500	0	0	0	0	0	0	0	0	0	0	0	0	808	0.248195122	3080	0.078734174
G6	500	0	0	0	0	0	0	0	0	0	0	0	0	2880	1.038956324	11200	0.336243195
G8	500	0	0	0	0	0	0	0	0	0	0	0	0	840	0.253424517	3830	0.09616096
G9	500	0	0	0	0	0	0	0	0	0	0	0	0	813	0.222554376	1870	0.042600812
G11	500	0	0	0	0	0	0	0	0	0	0	0	0	1570	0.51271887	4780	0.129908696
G12	500	0	0	0	0	0	0	0	0	0	0	0	0	2670	0.790160146	1840	0.045316083
G14	500	0	0	0	0	0	0	0	0	0	0	0	0	545	0.170217712	2860	0.074336987

Sample number	20cLTB4		13,14_dPGF2α		13,14_Dpge2		13,14_dPGD2		20_HETE		12_HETE		12_HETrE		5,6_diHET		19_HETE	
	peak area	concentration	peak area	concentration	peak area	concentration	peak area	concentration	peak area	concentration	peak area	concentration	peak area	concentration	peak area	concentration	peak area	concentration
A1	0	0	73900	50.11890995	0	0	0	0	0	0	108000	0.990692308	2730	0.079241988	1020	0.021184615	0	0
A2	0	0	49900	41.78041074	0	0	0	0	0	0	44800	0.507350427	1240	0.044435429	1540	0.039487179	0	0
A3	0	0	38700	30.25510469	0	0	0	0	0	0	128000	1.353491465	4930	0.164956772	1370	0.032799823	0	0
A4	0	0	58100	45.22624382	0	0	0	0	0	0	69800	0.734899018	2620	0.087287331	1870	0.044577861	0	0
A6	0	0	33400	23.05531638	0	0	0	0	0	0	92200	0.860822079	3040	0.08981187	1230	0.026001174	0	0
A8	0	0	48500	36.95803823	0	0	0	0	0	0	44700	0.460715212	1370	0.04468104	1400	0.0326707	0	0
A9	0	0	66800	50.68945083	0	0	0	0	0	0	15000	0.153953739	1110	0.036049533	1340	0.031139322	0	0
A11	0	0	53000	36.35352694	0	0	0	0	0	0	11500	0.106690654	221	0.006487816	2820	0.059235632	0	0
A12	0	0	45500	35.77745724	0	0	0	0	0	0	29800	0.316936455	1150	0.038701826	2710	0.065257525	0	0
A14	0	0	81300	64.0204156	0	0	0	0	0	0	19200	0.204497041	1200	0.040443127	4490	0.108277325	0	0
G1	0	0	58800	52.64436207	0	0	0	0	0	0	80300	0.972406702	3000	0.114955917	3830	0.105011424	0	0
G2	0	0	68400	58.81307786	0	0	0	0	0	0	12800	0.148862611	1840	0.067712861	3860	0.101640863	0	0
G3	0	0	73000	60.74666357	0	0	0	0	0	0	69900	0.786746107	3150	0.112187807	4140	0.105502596	0	0
G4	0	0	80000	71.6249824	0	0	0	0	0	0	16100	0.194965727	2290	0.087749684	5730	0.157105864	0	0
G6	0	0	182000	191.3677946	0	0	0	0	0	0	201000	2.858586762	7360	0.331215623	4600	0.148121646	0	0
G8	0	0	112000	98.48725295	0	0	0	0	0	0	15900	0.189111083	462	0.017387595	5830	0.156997881	0	0
G9	0	0	54500	43.48452746	0	0	0	0	0	0	19200	0.20720362	726	0.024791934	2470	0.060352941	0	0
G11	0	0	84300	80.24165627	0	0	0	0	0	0	9370	0.120634008	800	0.032591011	4970	0.144874494	0	0
G12	0	0	41400	35.71057648	0	0	0	0	0	0	21400	0.249671028	970	0.035809956	3540	0.093511068	0	0
G14	0	0	109000	99.2264385	0	0	0	0	0	0	15600	0.192080537	1040	0.040520032	3220	0.089767682	0	0

Sample number	15_HETE		9,10_DiHOME		9_HODE		13_HODE		15LXA4		AA		PGF2α		Resolvin D4		Resolvin D5	
	peak area	concentration	peak area	concentration	peak area	concentration	peak area	concentration	peak area	concentration	peak area	concentration	peak area	concentration	peak area	concentration	peak area	concentration
A1	2510	0.028506429	18600	0.017283506	22800	1.326292683	87100	1.290270186	0	0	557000	486.0164634	5300	0.180149644	0	0	0	0
A2	2210	0.030986772	241000	0.276471861	68400	4.912195122	202000	3.694271912	0	0	430000	463.2113821	6060	0.254299287	0	0	0	0
A3	3760	0.049225196	91100	0.097581571	22900	1.535573206	97400	1.66322874	0	0	479000	481.7944753	3690	0.144582064	0	0	0	0
A4	3430	0.044711621	42900	0.045754458	22000	1.46887357	58200	0.989561296	0	0	304000	304.4574308	1450	0.056569553	0	0	0	0
A6	4530	0.052364231	18400	0.017402201	31100	1.841333085	73900	1.114228818	0	0	318000	282.4166822	6740	0.233176552	0	0	0	0
A8	1810	0.023097111	297000	0.310088283	42300	2.764743765	121000	2.013992602	0	0	327000	320.5926281	3050	0.116484294	0	0	0	0
A9	1140	0.014486314	73600	0.076521006	23300	1.516506908	65000	1.077357425	0	0	133000	129.8468361	3020	0.114854609	0	0	0	0
A11	1350	0.015506592	36800	0.034584399	19300	1.13546915	52400	0.78506804	0	0	274000	241.8019796	3000	0.103131728	0	0	0	0
A12	3010	0.039634783	49200	0.053005985	26700	1.800763521	72000	1.236618958	0	0	609000	616.1039236	1990	0.078424455	0	0	0	0
A14	504	0.006646154	55900	0.060311688	22700	1.533208255	70400	1.210893454	0	0	357000	361.6885553	2000	0.078932944	0	0	0	0
G1	2350	0.03523338	29600	0.036310145	20900	1.604974644	70700	1.382608696	0	0	662000	762.5549384	1490	0.066859199	0	0	0	0
G2	2510	0.036141272	74900	0.088239159	20700	1.526636002	67200	1.262096052	0	0	564000	623.9294963	1840	0.079293208	0	0	0	0
G3	3070	0.042780894	61900	0.070575173	25700	1.834340865	80400	1.461372556	0	0	508000	543.8784977	11800	0.492132375	0	0	0	0
G4	2390	0.035833098	99900	0.12254674	29700	2.280753441	77500	1.515589447	0	0	617000	710.7196329	3010	0.135064556	0	0	0	0
G6	5120	0.090152824	36100	0.052007551	47700	4.301928531	95600	2.195637729	0	0	668000	903.675553	2760	0.145447716	0	0	0	0
G8	1520	0.022382959	48300	0.058193016	18600	1.402885718	65600	1.260001812	0	0	456000	515.8999091	3490	0.153811139	0	0	0	0
G9	2930	0.039148739	25100	0.027439343	20100	1.375566714	57800	1.007329193	0	0	403000	413.6965567	4600	0.183948582	0	0	0	0
G11	2010	0.032039098	69200	0.09024853	22000	1.796148909	65200	1.355580255	0	0	536000	656.4107831	3800	0.18128266	0	0	0	0
G12	1680	0.024267091	70200	0.082965086	34600	2.55988212	77400	1.458286346	0	0	771000	855.6368995	4690	0.202754136	0	0	0	0
G14	2010	0.030641419	95700	0.119364334	21000	1.6397119	68300	1.358084956	0	0	476000	557.5020462	4100	0.187061806	0	0	0	0

Sample number	volume (uL)	10_HDHA		11_HDHA		13_HDHA		14,15 EpETE		17,18 EpETE		5,6 diHETE		20hLTB4		LTB4	
		peak area	concentration	peak area	concentration	peak area	concentration	peak area	concentration	peak area	concentration	peak area	concentration	peak area	concentration	peak area	concentration
A1	500	0	0	0	0	0	0	0	0	0	0	367	0.245409813	3810	0.16178368	5890	0.135290369
A2	500	0	0	0	0	0	0	0	0	0	0	490	0.404517134	4880	0.255825915	7720	0.218919208
A3	500	0	0	0	0	0	0	0	0	0	0	500	0.385413019	5710	0.27949649	4810	0.127358278
A4	500	0	0	0	0	0	0	0	0	0	0	286	0.219507368	6260	0.305099345	4710	0.124173723
A6	500	0	0	0	0	0	0	0	0	0	0	325	0.221195691	3190	0.137869391	6370	0.148921863
A8	500	0	0	0	0	0	0	0	0	0	0	1060	0.796419196	5760	0.274816124	6980	0.180142839
A9	500	0	0	0	0	0	0	0	0	0	0	1000	0.748186393	4580	0.217600133	2360	0.060652336
A11	500	0	0	0	0	0	0	0	0	0	0	528	0.357086381	5630	0.241786268	2120	0.049249401
A12	500	0	0	0	0	0	0	0	0	0	0	2060	1.597106867	4990	0.245669462	2190	0.058322563
A14	500	0	0	0	0	0	0	0	0	0	0	500	0.388209921	10300	0.507829263	3300	0.088010866
G1	500	0	0	0	0	0	0	0	0	0	0	1400	1.235865643	7480	0.419303699	1130	0.034264736
G2	500	0	0	0	0	0	0	0	0	0	0	1000	0.847786483	7770	0.418303385	2080	0.060572507
G3	500	0	0	0	0	0	0	0	0	0	0	1760	1.44404564	7730	0.402746355	2750	0.077504456
G4	500	0	0	0	0	0	0	0	0	0	0	1030	0.909244009	9620	0.539264918	1900	0.057613273
G6	500	0	0	0	0	0	0	0	0	0	0	1360	1.409954358	3830	0.252144089	11100	0.395289111
G8	500	0	0	0	0	0	0	0	0	0	0	1160	1.00574683	9470	0.521392206	2340	0.069690289
G9	500	0	0	0	0	0	0	0	0	0	0	1400	1.101374382	5670	0.283252575	2370	0.064044377
G11	500	0	0	0	0	0	0	0	0	0	0	2710	2.543374324	7580	0.451745744	2990	0.096391315
G12	500	0	0	0	0	0	0	0	0	0	0	1200	1.020578577	4520	0.24411109	2320	0.067776465
G14	500	0	0	0	0	0	0	0	0	0	0	508	0.455966882	13700	0.78086153	1860	0.057346677

Sample number	5_HETE		PGJ2		15_DPGJ2		6_kPGF1α		14,15_EET		11,12_EET		8,9_EET		11_HETE		15_OXoETE	
	peak area	concentration	peak area	concentration	peak area	concentration	peak area	concentration	peak area	concentration	peak area	concentration	peak area	concentration	peak area	concentration	peak area	concentration
A1	1450	0.044336538	0	0	0	0	0	0	2140	0.054200708	3460	0.023804135	1590	0.050901342	0	0	0	0
A2	978	0.036918803	0	0	0	0	0	0	1400	0.043775811	3180	0.027009615	878	0.034700969	0	0	0	0
A3	2320	0.081773443	0	0	0	0	0	0	2200	0.064230956	5560	0.044094214	2120	0.078234532	0	0	0	0
A4	744	0.026111025	0	0	0	0	0	0	2930	0.08517576	2050	0.016187783	1470	0.054014039	0	0	0	0
A6	1970	0.061309454	0	0	0	0	0	0	1800	0.046401405	8920	0.062460951	670	0.021831036	0	0	0	0
A8	433	0.014876188	0	0	0	0	0	0	1800	0.051224023	4020	0.031075086	1570	0.056473117	0	0	0	0
A9	324	0.011084669	0	0	0	0	0	0	943	0.026723099	1660	0.01277816	1600	0.057310743	0	0	0	0
A11	473	0.014627443	0	0	0	0	0	0	900	0.023054048	1930	0.013429106	600	0.019426603	0	0	0	0
A12	607	0.021519064	0	0	0	0	0	0	1050	0.030833397	3090	0.024647659	1780	0.066068281	0	0	0	0
A14	657	0.023325444	0	0	0	0	0	0	1140	0.033524847	3370	0.026920118	842	0.031297883	0	0	0	0
G1	1220	0.049246002	0	0	0	0	0	0	1360	0.045472356	5140	0.046682788	709	0.029963719	0	0	0	0
G2	980	0.037990979	0	0	0	0	0	0	1400	0.044955191	2750	0.023986651	1330	0.053981429	0	0	0	0
G3	962	0.036092025	0	0	0	0	0	0	955	0.029678158	6010	0.050733306	950	0.03731626	0	0	0	0
G4	1340	0.054089871	0	0	0	0	0	0	1010	0.033769912	2570	0.023341394	1000	0.042261944	0	0	0	0
G6	1420	0.067316637	0	0	0	0	0	0	3020	0.118587364	15700	0.167461986	3980	0.19754019	0	0	0	0
G8	531	0.021051989	0	0	0	0	0	0	1500	0.04925919	2140	0.019089515	1400	0.058111886	0	0	0	0
G9	864	0.031080543	0	0	0	0	0	0	1900	0.056614263	1960	0.015864027	415	0.015630083	0	0	0	0
G11	340	0.014591093	0	0	0	0	0	0	1440	0.051188076	2780	0.02684332	800	0.035944896	0	0	0	0
G12	1110	0.043167421	0	0	0	0	0	0	557	0.017942614	3060	0.026775468	970	0.039495097	0	0	0	0
G14	675	0.027703924	0	0	0	0	0	0	1650	0.056094316	2840	0.026226381	1400	0.060159452	0	0	0	0

Sample number	8_HETE		13_OXoODE		9_OXoODE		12,13_Epome		PGE2		PGD2		Resolvin D1	
	peak area	concentration	peak area	concentration	peak area	concentration	peak area	concentration	peak area	concentration	peak area	concentration	peak area	concentration
A1	2940	0.075804324	13900	0.309827103	17000	0.620586735	62300	1.183948207	47100	0.440523529	1200	0.081771429	0	0
A2	1020	0.032468468	22400	0.616407061	48700	2.194812925	148000	3.472332891	43600	0.503442266	1090	0.091698413	0	0
A3	1540	0.045771789	24600	0.632077352	22100	0.929985885	78600	1.721859536	47200	0.508886252	1100	0.086405928	0	0
A4	1400	0.041431618	20300	0.519347269	17800	0.745814423	50400	1.099340943	67900	0.728912144	997	0.07797811	0	0
A6	1110	0.029129771	15700	0.356181779	17800	0.661364699	55300	1.069639001	21500	0.20466996	890	0.061727372	0	0
A8	1180	0.034185241	24600	0.616097868	24100	0.988508943	94900	2.026379874	46400	0.487614012	734	0.056198716	0	0
A9	1200	0.034618787	19400	0.483827201	13000	0.530983302	51300	1.090802106	56800	0.594402304	780	0.05947013	0	0
A11	317	0.008266457	18600	0.419305977	19300	0.712564824	45600	0.876441642	44900	0.424725218	800	0.055134549	0	0
A12	873	0.026097673	21900	0.565965055	23400	0.99039929	53500	1.178797852	32700	0.354598465	797	0.06296795	0	0
A14	702	0.021016216	17200	0.445147376	17500	0.741758242	58400	1.288630095	25000	0.271493213	983	0.077775824	0	0
G1	877	0.029851325	25900	0.762117146	20300	0.978288543	50200	1.259405941	20500	0.2531159	576	0.051815559	0	0
G2	563	0.018404146	20400	0.576494809	28500	1.319043307	60600	1.460084227	15900	0.188541065	910	0.078618067	0	0
G3	1010	0.03195291	18100	0.495023221	20900	0.936143108	59500	1.387407426	21600	0.247881631	549	0.045902366	0	0
G4	1000	0.034037998	28400	0.835680577	27100	1.305991109	59600	1.495230957	33300	0.411158998	3120	0.28066761	0	0
G6	2840	0.113528598	21300	0.736079113	39800	2.252562886	74200	2.186194756	21200	0.307414501	1490	0.157415282	0	0
G8	363	0.012135512	22600	0.653157424	17700	0.837783217	49700	1.224632749	27400	0.332279531	812	0.071743274	0	0
G9	854	0.025905119	23500	0.61624519	21800	0.936248499	44500	0.99491446	39500	0.434636678	1000	0.080168067	0	0
G11	749	0.027104637	24500	0.766453517	28900	1.480698174	44900	1.197584399	28800	0.378055728	880	0.084162406	0	0
G12	279	0.009149349	22700	0.643531492	27800	1.290736835	55300	1.336623617	29400	0.3497316	1410	0.122202135	0	0
G14	777	0.026891275	21600	0.646252274	23100	1.131903164	52100	1.329003449	43200	0.542345045	1710	0.156408437	0	0

Supplemental Table 5 Baseline characteristcs and laboratory findings of gout flare and gout remission in scRNA-seq study

Patient number	P1		P2		P3	
Age, years	53		53		23	
Gender	male		male		male	
Height, cm	177		178		175	
Weight, kg	69.5		80.0		85.0	
BMI,kg/m^2	22.18		25.25		27.76	
Medical illness	ypertension,nephrolithiasis,gou		ypertension,nephrolithiasis,fatty liver,renal cyst,gou		gout, fatty liver	
Disease stage	acute flare	remission	acute flare	remission	acute flare	remission
Dual source CT, tophi	positive		positive		positive	
Duration of gout	20		20		2	
Use of urate-lowering therapy at the time of their entry flare	yes		yes		yes	
se of anti-inflammatory agents, including colchicine and anti-IL1 therapy, at the time of their flare (besides steroids and NSAIDs)	No		No		No	
Red blood cell count, X10^12/L, (4.30-5.80)	4.03	4.85	4.40	4.27	4.82	4.97
Haemoglobin, g/l, (130-175)	128.00	143.00	144.00	138.00	140.00	147.00
White blood cell count, X10^9/L, (3.50-9.50)	9.21	7.80	10.90	6.68	7.09	6.91
Platelet count, X10^9/L, (125-350)	338.00	329.00	179.00	195.00	310.00	221.00
Neutrophil percentage, %, (40-75)	63.90	57.10	66.30	53.90	64.10	62.40
Lymphocyte percentage, %, (20-50)	23.00	31.20	22.20	34.40	28.10	28.90
Monocyte percentage, %, (3-10)	8.70	6.70	9.70	8.70	5.50	6.00
Eosinophils percentage, %, (0.4-8)	3.50	4.50	1.50	2.40	1.90	2.40
Basophils percentage, %, (0-1)	0.90	0.50	0.30	0.60	0.40	0.30
C-reactive protein, mg/L, (<8.2)	6.97	2.43	12.40	2.59	38.82	3.95
Alanine aminotransferase, IU/L, (9-50)	16.70	14.50	39.00	24.50	19.90	6.80
Aspartate aminotransferase, IU/L, (15-40)	18.20	14.10	52.30	18.20	21.20	25.10
Total protein, g/L, (65-85)	76.70	72.50	78.60	71.50	81.30	76.60
Albumin, g/L(40-55)	43.30	41.10	45.80	43.20	48.00	49.50
Fasting Blood Glucose, mmol/L, (3.9-6.1)	4.50	4.61	5.00	5.30	4.70	4.80
Urea, mmol/L, (2.76-8.07)	6.25	5.90	8.11	5.40	4.19	3.54
esimate Glomerular Filtration Rate, ml/min/L, (>90)	87.94	90.23	145.95	91.41	115.68	124.45
Serum Creatine, umol/L, (57-97)	87.00	84.00	81.20	83.00	82.00	72.00
Uric acid, umol/L, (208-428)	486.00	512.00	335.00	522.00	533.00	646.00
Total Cholesterol, mmol/L, (<5.2)	2.95	3.76	2.68	3.33	4.66	4.80
Triglyceride, mmol/L, (<1.7)	1.93	2.59	1.97	2.92	1.19	1.30
High-Density Lipoprotein, mmol/L, (1.04-1.55)	1.16	1.10	1.20	1.70	0.69	1.01
Low-Density Lipoprotein, mmol/L, (<3.34)	0.93	2.09	0.53	1.20	3.28	3.15

Supplemental Table 6 Baseline characteristcs and laboratory findings of gout flare and gout remission in an independent validation cohort

sample ID	Group	Gender	Age, years	Height, cm	Weight, kg	BMI,kg/m^2	Red blood cell count, X10^12/L	Haemoglobin, g/l	White blood cell count, X10^9/L	Platelet count, X10^9/L	Neutrophil percentage, %	Lymphocyte percentage, %	Monocyte percentage, %	Eosinophils percentage, %	Basophils percentage, %	lanine aminotransferase, IU/L	Irea, mmol/L	Serum Creatine, umol/L	Jric acid, umol/L
AG1	Acute flare	male	72	168	65	23.03	3.14	96	12.13	218	86.2	7.1	6.4	0.2	0.1	15.7	15.2	219	574
A2	Acute flare	male	43	175	93	30.37	4.89	150	12.77	250	76.9	12.4	8.4	2.2	0.1	79.5	3.41	75.6	548.1
AG4	Acute flare	male	61	172	73	24.68	4.86	150	5.99	188	56.9	33.6	5.7	2.8	1	40.3	5.8	80	564
AG7	Acute flare	male	51	175	83	27.10	5.55	178	5.53	171	57.1	32.5	7.8	2.2	0.4	24.4	5.1	102	397
A8	Acute flare	male	32	175	75	24.49	5.23	164	5.63	205	60.9	26.9	8.9	3	0.3	34	4.9	74	630
A9	Acute flare	male	34	173	76	25.39	5.62	173	11.15	259	74.1	20.4	5	0.3	0.2	66.7	7.68	101.9	437.4
A10	Acute flare	male	26	175	75	24.49	5.17	163	13.32	250	78.8	15	5.6	0.5	0.1	38.2	4.73	92.9	565.3
A13	Acute flare	male	46	175	80	26.12	5.61	164	8.12	253	69.7	17.5	6.2	6.2	0.4	14.3	5.3	91	543
AG12	Acute flare	male	55	170	65	22.49	5.07	154	6.58	203	72.5	20.2	0.43	0.3	0.4	18.2	4.6	116	431
AG11	Acute flare	male	34	173	90	30.07	5.41	165	16.39	351	80.1	12.5	5.1	2.1	0.2	27.4	4.43	82.7	630.8
AG14	Acute flare	male	49	173	81	27.06	5.04	148	16.28	244	62.8	29.9	6.3	0.9	0.1	49	4.18	86.6	450.6
AG16	Acute flare	male	53	175	86	28.08	5.17	166	8.21	212	53.1	35.9	8.9	1.9	0.2	34.5	4.64	66.7	461
AG21	Acute flare	male	34	182	90	27.17	5.51	157	6.62	368	60.4	29.2	8.2	1.8	0.4	31	4.57	79.5	582.3
AG23	Acute flare	male	58	176	65	20.98	5.03	155	9.84	235	78.8	12.4	7.6	1	0.2	19.4	6.01	125.7	545.1
AG24	Acute flare	male	59	178	75	23.67	5.74	139	9.22	198	63.1	27.8	8.2	0.5	0.4	9.8	4.72	69.6	452.5
AG33	Acute flare	male	43	170	65	22.49	5.17	155	10.35	261	76	19	4	0.8	0.2	69.5	2.42	54.4	477.6
AG36	Acute flare	male	33	180	81	25.00	5.27	168	7.32	219	69.6	23.7	6.2	0.3	0.2	27.7	4.14	82.9	485.8
AG40	Acute flare	male	35	172	83	28.06	5.56	164	10.35	218	65	26.8	7.7	0.4	0.1	33.2	5	89	263
AG41	Acute flare	male	54	164	74.5	27.70	5.18	155	7.54	197	74.3	18.6	6.4	0.5	0.2	47.1	3.84	78.3	177.2
AG42	Acute flare	male	40	179	86	26.84	4.88	151	4.84	338	54.6	37.2	4.7	2.6	0.9	27.1	4.52	74.8	414.4
AG43	Acute flare	male	56	165	68.5	25.16	3.8	100	7.09	336	82.9	13	3.5	0.3	0.3	4	9.6	193	609
AG44	Acute flare	male	33	175	72	23.51	5.24	153	7.08	260	58.4	33.6	5.6	2	0.4	32.3	4.55	77.4	535.9
AG49	Acute flare	male	44	172	75	25.35	5.25	158	9.09	329	69.2	23.1	5.3	1.9	0.5	35.8	4.93	111.9	524.7
AG50	Acute flare	male	38	175	75	24.49	4.75	154	8.77	286	71.5	22.8	4.8	0.6	0.3	24.2	5.54	108.2	474.2
A52	Acute flare	male	35	178	78	24.62	5.02	158	11.83	290	63.6	27	7.9	1.2	0.3	25.6	4.1	64	687
AG53	Acute flare	male	42	170	75	25.95	4.75	151	5.78	376	59.9	30.7	7.6	1.3	0.5	89.6	5.76	82.3	581.8
AG54	Acute flare	male	62	186	90	26.01	5.3	165	8.69	186	60.9	31.7	5.5	1.7	0.2	36.5	4.09	61.7	508.3
AG55	Acute flare	male	58	182	90	27.17	5	149	7.58	208	75.3	17.8	5.6	1.1	0.2	31.7	5.91	47.5	606.6
AG56	Acute flare	male	50	170	90	31.14	4.5	143	6.13	216	70.5	24.3	3.7	1.3	0.2	23.1	4.33	52	528.5
AG58	Acute flare	male	34	178	85	26.83	4.85	156	8.37	219	57.1	23.9	3.9	14.5	0.6	20.3	5.91	76.4	453.5
AG59	Acute flare	male	40	180	100	30.86	5.22	159	10.05	270	54.8	34.5	6.4	4	0.3	88.2	5.5	70.7	540
P1	Acute flare	male	53	177	69.5	22.18	4.03	128	9.21	338	63.9	23	8.7	3.5	0.9	16.7	6.25	87	86
P2	Acute flare	male	53	178	80	25.25	4.4	144	10.9	179	66.3	22.2	9.7	1.5	0.3	39	8.11	81.2	335
AG3	Acute flare	male	38	172	94	31.77	5.06	155	7.56	233	56.2	37.4	5	1.3	0.1	78	3	83	549
P3-A	Acute flare	male	23	175	85	27.76	4.82	140	7.09	310	64.1	28.1	5.5	1.9	0.4	19.9	4.19	82	533
AG5	Acute flare	male	27	178	98	30.93	4.96	155	8.07	275	66.9	25.3	6.4	1.1	0.3	195.8	4.74	96	798
AG6	Acute flare	male	52	170	70	24.22	4.68	156	7.35	325	47.8	36.4	6.8	8.3	0.7	21.9	5.59	81	506
AG8	Acute flare	male	50	169	70	24.51	4.22	115	6.96	275	57.1	28.3	8.3	6	0.3	5.9	4.26	105	628
AG9	Acute flare	male	31	173	75	25.06	4.69	144	5.26	205	58.7	25.3	6.6	8.7	0.7	8	5.48	87	464
AG10	Acute flare	male	47	170	80	27.68	5.4	157	7.16	268	63	26.4	8.2	2	0.4	42.4	3.68	76	302
AG13	Acute flare	male	64	170	80	27.68	3.74	116	5.9	207	59.6	29	7.5	3.6	0.3	22.6	6.51	111	860
AG15	Acute flare	male	68	170	71	24.57	4.93	149	5.98	288	49	37	10.5	2.8	0.7	10.8	6.18	82	546
AG19	Acute flare	male	64	170	66	22.84	3.36	92	7.53	310	67.1	24.6	5.8	2.4	0.1	37	13.9	173	510
AG17	Acute flare	male	35	165	105	38.57	5.31	154	8.71	415	57.8	30.3	8.4	2.6	0.9	78.7	4.4	74	723
AG20	Acute flare	male	57	176	71	22.92	4.33	134	4.37	188	56.7	33.2	8	1.6	0.5	9.3	4.7	128	391
AG18	Acute flare	male	55	168	78	27.64	4.78	146	6.65	245	47.6	40.2	8.1	3.3	0.8	30.1	2.9	63	315
P3-R	Remission	male	23	175	85	27.76	4.97	147	6.91	221	62.4	28.9	6	2.4	0.3	6.8	3.54	72	646
G2	Remission	male	43	175	93	30.37	5.13	157	7.51	213	60.7	26.1	6.6	6.1	0.5	67	5.4	73	467
G7	Remission	male	77	165	82	30.12	4.38	148	5.98	167	55.9	30.8	10.4	2.2	0.7	64.1	5.9	77	363
G8	Remission	male	32	175	75	24.49	8.01	169	8.01	233	61.5	30.2	5.6	2.4	0.3	25.1	4.2	80	454
G9	Remission	male	34	173	76	25.39	5.4	164	6.36	223	63.9	28.8	6.5	0.5	0.3	42.5	4.7	86	469
G10	Remission	male	46	175	80	26.12	5.46	151	7.86	237	65	24	5	5.5	0.5	24.8	4.4	78	369
G13	Remission	male	55	170	65	22.49	5.51	164	5.97	185	56.7	36.1	5.7	1.1	0.4	24.2	6.7	129	632
RG42	Remission	male	40	179	86	26.84	4.9	149	6.2	333	48.6	42.8	5.3	2.8	0.5	60.7	4.8	77	453
RG43	Remission	male	56	165	68.5	25.16	4.18	112	7.47	185	75.7	18.7	5.3	0.4	0.1	5.7	14	220	596
G52	Remission	male	35	178	78	24.62	4.68	148	8.88	263	57.6	32	8	2.1	0.3	21	3.3	62	645
G22	Remission	male	45	180	76	23.46	5.21	159	5.97	273	59.1	35.8	4.4	0.7	0	21.9	4.67	82.6	477.3
G23	Remission	male	53	181	95	29.00	5.36	159	5.87	229	59.8	32.2	6.6	0.9	0.5	24.6	6.7	87	732
RG28	Remission	male	33	190	95	26.32	5.4	157	5.26	255	47	36.9	11.8	3	1.3	15.5	5.4	79	550
RG29	Remission	male	52	170	70	24.22	4.75	153	5.23	226	62	27.2	7.1	3.1	0.6	36	5.7	93	521
RG30	Remission	male	27	168	88	31.18	4.97	154	6.06	247	49.1	41.8	6.5	2.1	0.5	86.8	3.3	90	397
RG31	Remission	male	76	172	75	25.35	3.25	111	5.66	157	67.9	22.8	7.4	1.4	0.5	12.5	10.4	122	486
RG9	Remission	male	71	165	80	29.38	4.53	135	10.07	309	49.2	40.2	7	2.9	0.7	11.5	6.9	121	372
RG11	Remission	male	45	174	70	23.12	5.13	146	5.58	309	58.6	33.4	5.7	1.7	0.6	21.5	4.6	92	403
RG14	Remission	male	55	182	86	25.96	5.04	164	7.71	212	75.9	17.2	5.8	1	0.1	22.5	4.1	104.6	583
RG15	Remission	male	68	174	85	28.08	4.16	128	5.22	234	64.9	25.1	7.5	1.9	0.6	23.6	7.4	141	446
RG39	Remission	male	54	172	80	27.04	5.85	165	8.01	188	64.5	27.7	5.3	2.1	0.4	40.3	5.1	72	407
RG1	Remission	male	56	177	78.8	25.15	4.63	149	7.2	175	58.4	27.1	10.4	3.3	0.8	19.3	6	74	535
RG2	Remission	male	56	178	73.4	23.17	4.15	136	6.27	160	52.9	32.9	10.5	2.9	0.8	37	6.6	81	411
RG3	Remission	male	59	168	75	24.49	6.26	130	4.42	217	74.4	16.6	7.7	1	0.3	21	9.5	120	458
RG4	Remission	male	56	173	65	21.72	5.1	155	5.81	189	51.7	38.5	5.5	3.5	0.8	22.7	4	95	564
RG5	Remission	male	65	169	74	25.91	4.03	108	4.05	135	50.1	39.5	6.2	3.5	0.7	14.1	5.3	112	605
RG6	Remission	male	69	170	71	24.57	5.07	154	7.22	295	52.6	36.5	8.1	2.3	0.5	25.4	5.8	92	576
RG7	Remission	male	28	178	97	30.61	5.31	156	6.8	240	61.5	30.6	6.9	0.9	0.1	77.7	3.2	119.6	484
RG10	Remission	male	57	187	82	23.45	5.87	161	5.03	184	55.1	33.8	8.7	1.6	0.8	25.1	7.1	99.5	244
RG12	Remission	male	64	168	55	19.49	3.63	113	4.54	221	52.2	32.8	9	5.3	0.7	29.6	23.9	176	421
RG13	Remission	male	52	168	76	26.93	4.31	139	7.92	210	76.6	16.9	4.9	1.3	0.3	28.4	4.2	99	540
R14	Remission	male	49	173	81	27.06	5.25	154	13.17	288	51.5	41.5	4.9	1.6	0.5	71.4	4.1	83	463.2
RG16	Remission	male	53	175	86	28.08	5.41	171	7.44	193	51.8	41.7	4.8	1.5	0.2	17.7	4	103.2	736
RG19	Remission	male	69	170	75	25.95	3.72	105	6.92	332	67.7	19.8	8.2	3.9	0.4	9.7	8.8	137	367
RG20	Remission	male	71	165	71	26.08	4.04	124	7.39	104	69	23.1	7	0.8	0.1	34.3	7.8	122	141
RG21	Remission	male	49	181	75	22.89	4.95	151	9.5	233	67.6	22.7	6.7	2	1	23.6	5.2	92	353
RG22	Remission	male	43	170	95	32.87	5.12	150	8.88	311	66.1	24	7.4	2.2	0.3	57.8	5.7	72	586
RG23	Remission	male	65	170	71	24.57	4.89	148	5.6	249	54.9	36	7.2	1.6	0.3	11.2	5	83	470
RG56	Remission	male	42	164	75	27.89	4.76	148	9.57	361	59.5	25	5.6	9.2	0.7	41.2	5.9	77	389
RG32	Remission	male	44	174	76	25.10	4.8	142	3.63	182	54.5	32	8.1	4.4	1	21.9	4.43	110	604
RG33	Remission	male	49	170	69	23.88	5.37	167	5.8	166	64.6	1.41	7.2	3.4	0.5	27.7	3.96	65	454
RG34	Remission	male	33	175	85	27.76	5.29	170	5.54	172	52.8	30.4	6.5	9.9	0.4	50.6	3.15	80	613
RG35	Remission	male	41	175	95	31.02	4.71	148	5.84	206	64.9	27.2	6.1	1.4	0.4	20.3	7.8	103	517
RG36	Remission	male	34	168.5	73.5	25.89	4.28	131	6.74	260	47.2	42.3	7.7	1.9	0.9	44.8	4.1	101	489
RG37	Remission	male	64	168	60	21.26	3.97	119	5.81	284	60.7	27	5.2	6.2	0.9	79.7	8.2	104	516
RG38	Remission	male	65	172	68	22.99	4.41	143	6.96	174	59	30	7.3	3.4	0.3	13.8	3.1	74	449
RG49	Remission	male	56	171	76	25.99	4.23	140	5.01	151	50.3	35.1	11	3.2	0.4	28	4	78	456
RG40	Remission	male	39	163	56	21.08	7.07	142	7.55	310	73.8	21.8	3.3	0.9	0.2	57.5	5.8	78	435
RG41	Remission	male	55	168	78	27.64	4.92	147	7.53	242	62.1	27.8	6.81	2.6	0.4	35.1	5.7	94	494
RG44	Remission	male	63	176	82	26.47	5.02	148	6.78	212	57.1	27.4	11.4	3.1	1	35.9	5	87	560

Supplemental Table 7 Baseline characteristcs and laboratory findings of gout flare and gout remission in LC-MS/MS cohort

Gender	male		male		male		male		male		male		male		male		male		male	
Age, years	72		43		74		61		66		32		34		46		55		42	
Height, cm	168		175		165		172		180		170		173		175		170		180	
Weight, kg	65		93		80		73		80		73		76		80		65		105	
BMI,kg/m^2	23.03		30.67		29.38		24.68		24.69		24.39		26.39		26.12		21.22		32.41	
Dual source CT, tophi	positive		positive		positive		positive		positive		positive		positive		positive		positive		positive	
Sample Number	A1	G1	A2	G2	A3	G3	A4	G4	A6	G6	A8	G8	A9	G9	A11	G11	A12	G12	A14	G14
Disease stage	acute flare	remission	acute flare	remission	acute flare	remission	acute flare	remission	acute flare	remission	acute flare	remission	acute flare	remission	acute flare	remission	acute flare	remission	acute flare	remission
Red blood cell count, X10^12/L, (4.30-5.80)	3.14	3.06	4.89	4.79	3.84	3.62	4.86	4.57	4.4	4.68	5.23	5.56	5.62	3.64	5.61	5.46	6.58	5.51	5.02	4.83
Haemoglobin, g/l, (130-175)	96	92	150	150	123	118	150	143.78	126	136	164	169	173	164	164	151	154	164	152	150
White blood cell count, X10^9/L, (3.50-9.50)	12.13	3.48	12.77	7.73	7.57	6.88	5.99	6.73	5.12	8.52	5.63	8.01	11.15	6.36	8.12	7.86	6.58	5.97	9.18	5.77
Platelet count, X10^9/L, (125-350)	218	256	250	228	173	152	188	237.56	341	332	205	233	259	223	253	237	203	185	340	292
Neutrophil percentage, %, (40-75)	86.2	59.2	76.9	62.6	61.2	59	56.9	54.89	46.9	59.7	60.9	61.5	74.1	63.9	69.7	65	72.5	3.38	66	59.7
Lymphocyte percentage, %, (20-50)	7.1	23.9	12.4	25.1	27.5	31.5	33.6	24.79	41	29.8	26.9	30.2	20.4	28.8	17.5	24	20.2	2.15	24.1	27.7
Monocyte percentage, %, (3-10)	6.4	10.6	8.4	6.5	10.3	7.6	5.7	6.54	8.6	6.7	8.9	5.6	5	6.5	6.2	5	6.6	0.34	9	10
Eosinophils percentage, %, (0.4-8)	0.2	5.7	2.2	5.4	0.5	1.5	2.8	2.39	2.9	3.4	3	2.4	0.3	0.5	6.2	0.43	0.3	0.07	0.8	2.1
Basophils percentage, %, (0-1)	0.1	0.6	0.1	0.4	0.5	0.4	1	0.33	0.6	0.4	0.3	0.3	0.1	0.3	0.4	0.04	0.4	0.03	0.1	0.5
C-reactive protein, mg/L, (<8.2)	211	16.5	54.64	6.84	139	3.8	3	4.94	18.4	8.72	11.63	0.9	3.97	3.64	2.67	0.74	31.02	1.18	12.88	2.13
Alanine aminotransferase, IU/L, (9-50)	15.7	42.1	79.5	73.6	59.3	25.6	40.3	35.3	19.3	11.8	34	46.3	66.7	42.5	27.4	33.4	18.2	24.2	70.1	67.5
Serum Creatine, umol/L, (57-97)	219	126	75.6	76	106	94	80	101	76	82	74.6	80	101.9	86	91	77	116	129	83.9	73
Uric acid, umol/L, (208-428)	574	463	548.1	413	671	461	564	503	491	410	507.3	454	437.4	469	543	316	431	632	508.4	555

Supplemental Table 8 Antibodies for cell staining required by flow cytometry

Celltype	Marker
Leukocyte	$CD45^+$
Myeloid cell	$CD45^+CD11b^+$
Classical monocyte	$CD45^+CD11b^+CD14^+CD16^-$
Non-classical monocyte	$CD45^+CD11b^+CD16^+CD14^-$
HLA-DQA1$^+$ classical monocyte	$CD45^+CD11b^+CD14^+CD16^-HLA\text{-}DQA1^+$
HLA-DQA1$^+$ non-classical monocyte	$CD45^+CD11b^+CD16^+CD14^-HLA\text{-}DQA1^+$
T cells	$CD45^+CD3^+$
CD4$^+$ T cells	$CD45^+CD3^+CD4^+$
Th1 cells	$CD45^+CD3^+CD4^+CXCR5^-CXCR3^+CCR4^-$
Th2 cells	$CD45^+CD3^+CD4^+CXCR5^-CXCR3^-CCR4^+$
Th17 cells	$CD45^+CD3^+CD4^+CXCR5^-CXCR3^-CCR4^-CCR6^+$
Treg cells	$CD45^+CD3^+CD4^+CD25^+CD127^-$

Supplemental Table 9 the source for commercial antibodies

Antibody	source	catalog number
CD45-FITC	BD Pharmingen	555482
Fixable viability stain 780	BD Pharmingen	565388
CD11B-PerCP-Cy5.5	BioLegend	393106
CD14-APC	BD Pharmingen	555399
CD16-PE-Cy7	BD Pharmingen	560716
HLA-DQA1-PE	Novus	NBP3-08747PE
CD3-PerCP-Cy5.5	BD Pharmingen	560835
CD4-APC-H7	BD Pharmingen	560158
CXCR5-AF647	BD Pharmingen	558113
CXCR3-AF488	BD Pharmingen	558047
CCR4-BV421	BD Pharmingen	562579
CCR6-BV510	BD Pharmingen	563241
CD25-PE	BD Pharmingen	555432
CD127-BV421	BD Pharmingen	562436
